# The genomic landscape of Epstein-Barr virus-associated pulmonary lymphoepithelioma-like carcinoma

**DOI:** 10.1038/s41467-019-10902-w

**Published:** 2019-07-16

**Authors:** Shaodong Hong, Dongbing Liu, Shuzhen Luo, Wenfeng Fang, Jianhua Zhan, Sha Fu, Yaxiong Zhang, Xuan Wu, Huaqiang Zhou, Xi Chen, Gang Chen, Zhonghan Zhang, Qiufan Zheng, Xiaobo Li, Jinghao Chen, Xingmin Liu, Mengyue Lei, Chen Ye, Jian Wang, Huanming Yang, Xun Xu, Shida Zhu, Yunpeng Yang, Yuanyuan Zhao, Ningning Zhou, Hongyun Zhao, Yan Huang, Lanjun Zhang, Kui Wu, Li Zhang

**Affiliations:** 1 Department of Medical Oncology, Sun Yat-sen University Cancer Center, 510060 Guangzhou, China; 2 State Key Laboratory of Oncology in South China, 510060 Guangzhou, China; 3 Collaborative Innovation Center for Cancer Medicine, 510060 Guangzhou, China; 4 BGI-Shenzhen, 518083 Shenzhen, China; 5 China National GeneBank-Shenzhen, BGI-Shenzhen, 518120 Shenzhen, China; 6 Department of Pathology, Sun Yat-Sen Memorial Hospital, Sun Yat-Sen University, 510120 Guangzhou, China; 7 Guangdong Provincial Key Laboratory of Malignant Tumor Epigenetics and Gene Regulation, 510120 Guangzhou, China; 8 Peking University Shenzhen Hospital, 518036 Shenzhen, China; 9 BGI Education Center, University of Chinese Academy of Sciences, 518083 Shenzhen, China; 10 Department of Thoracic Surgery, Sun Yat-sen University Cancer Center, 510060 Guangzhou, China

**Keywords:** Cancer genomics, Lung cancer

## Abstract

Pulmonary lymphoepithelioma-like carcinoma (LELC) is a rare and distinct subtype of primary lung cancer characterized by Epstein-Barr virus (EBV) infection. Herein, we reported the mutational landscape of pulmonary LELC using whole-exome sequencing, targeted deep sequencing and single-nucleotide polymorphism arrays. We identify a low degree of somatic mutation but widespread existence of copy number variations. We reveal predominant signature 2 mutations and frequent loss of type I interferon genes that are involved in the host-virus counteraction. Integrated analysis shows enrichment of genetic lesions affecting several critical pathways, including NF-κB, JAK/STAT, and cell cycle. Notably, multi-dimensional comparison unveils that pulmonary LELC resemble NPC but are clearly different from other lung cancers, natural killer/T-cell lymphoma or EBV-related gastric cancer in terms of genetic features. In all, our study illustrates a distinct genomic landscape of pulmonary LELC and provides a road map to facilitate genome-guided personalized treatment.

## Abstract (web summary)

The rare lung cancer subtype pulmonary lymphoepithelioma-like carcinoma is linked to Epstein-Barr virus infection. Here, the authors provide a mutational landscape for this cancer, showing a low burden of somatic mutations and high prevalence of copy number variations.

## Introduction

Pulmonary lymphoepithelioma-like carcinoma (LELC) is a rare subtype of primary lung cancer that histologically resembles undifferentiated nasopharyngeal carcinoma (NPC)^1^. First reported in 1987, pulmonary LELC has been recognized to be closely related to Epstein–Barr virus (EBV) infection^2,3^. In the 2015 World Health Organization (WHO) Classification of Lung Tumors, pulmonary LELC was moved from large cell carcinoma to other and unclassified carcinomas^4^. Compared with other types of lung cancers, pulmonary LELC has distinct clinicopathological features. It preferentially affects Asian non-smokers of younger age and has intensive tumor infiltrating immune cells^5^. Driver mutations such as epidermal growth factor receptor (*EGFR*) mutation and anaplastic lymphoma kinase (*ALK*) rearrangement are rarely detected in pulmonary LELC^6^. Palliative chemotherapy has been the major approach for metastatic disease, though immunotherapy singe-agent or in combination with chemotherapy might also be effective in patients with pulmonary LELC, which is currently categorized as non-small-cell lung carcinoma^7–9^. Due to the lack on the genomic data to date, the pathogenesis, rational histology classification and optimal treatment of pulmonary LELC remains not fully defined. Novel therapeutic agents are urgently needed to further improve of the survival of patients suffering from this lethal disease.

Based on the pathological characteristics of pulmonary LELC and its strong association with EBV infection, we hypothesize that pulmonary LELC is distinct from other types of lung cancers but similar to NPC in terms of genomic aberrations. In this study, we report the genomic landscape of a large cohort of pulmonary LELC by whole-exome sequencing (WES, *n* = 30), targeted deep sequencing (TDS, *n* = 61), and single-nucleotide polymorphism (SNP) array analysis (*n* = 46) (Supplementary Fig. 2a). We comprehensively compared the genomic profiles of pulmonary LELC with other relevant cancer types to further enhance the understanding of this unclassified carcinomas of the lung. Here, we identify key genetic lesions affecting pulmonary LELC. Integrative analyses through genomic data furthermore highlight pathways that may play critical roles in driving tumor progression. Multidimensional comparative study shows that pulmonary LELC is distinct from other primary lung cancers but share similarities with NPC, in terms of genomic features. These data provide important insights into the pathogenesis of pulmonary LELC and a road map to inform genome-guided personalized treatment for patients suffering from this rare tumor.

## Results

### Clinicopathological features

Paired fresh-frozen tumor tissue and adjacent normal tissue, and formalin-fixed, paraffin-embedded (FFPE) tissues were collected from a large cohort of 91 pathologically confirmed pulmonary LELC patients (Supplementary Figs. 1 and 2a, Supplementary Data 1 and 2). Among the patients, 43 (47%) were male, 17 (19%) had a history of smoking and the median age at diagnosis was 52.5 years (range: 27–71). The majority (56%) of the patients were diagnosed at an early stage (stage I, 31 patients; stage II, 21 patients) whose disease-free survival (DFS) and overall survival (OS) were significantly better than those at stage III–IV (Supplementary Fig. 2b).

### Somatic aberrations of pulmonary LELC

**Fig. 1:**
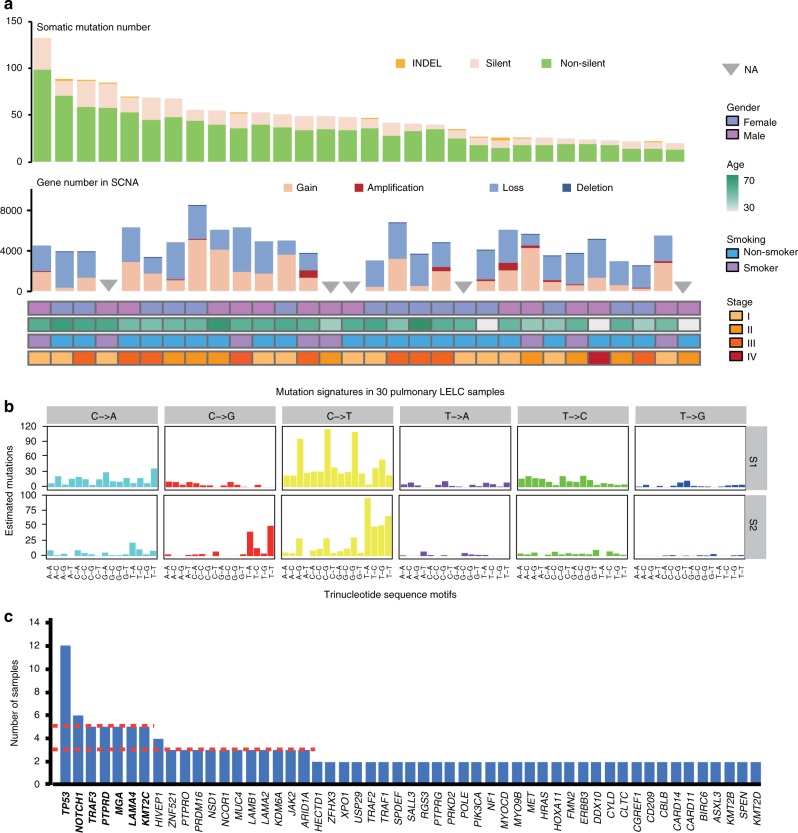
Somatic mutations and copy number alterations in pulmonary LELC. **a** The number of somatic mutations and copy number altered genes for each pulmonary LELC samples in the discovery cohort. Gender, age, smoking status, and tumor stages are listed at the bottom according to the samples. **b** Signatures are displayed according to the 96-substitution classification, with *x*-axis showed mutation types and *y*-axis showed the estimated mutations of each mutation type, which are identified by a Bayesian NMF algorithm. **c** Frequently mutated genes in the discovery cohort and validation cohort. The two red dashed line denote three and five mutated patients, respectively. Genes mutated in more than five patients are labeled with bold font. LELC, lymphoepithelioma-like carcinoma; SCNA, somatic copy number aberration; NA, not applicable; INDEL, insertion and deletion

We analyzed the WES data of 30 cases with a mean coverage of 100× (discovery cohort; Supplementary Data 3, Supplementary Fig. 3a) and identified 1461 somatic mutations including 1055 non-silent, 392 silent and 14 short insertions and deletions (Fig. 1a and Supplementary Data 4), revealing a low mutation rate (median: 1.2 mutations per megabase [Mb]; Supplementary Fig. 4). The predominant somatic mutation type was C:G > T:A transitions and C:G > G:C transversions (Supplementary Fig. 5). Two independent and stable mutational signatures were then identified (Fig. 1b and Supplementary Fig. 6). Signature 1 was reported to be positively correlated with age and was universally present in numerous cancer types^10^. Signature 2 was characterized primarily by C > T and C > G mutations at TpCpN trinucleotides and was attributed to the overactivity of the AID/APOBEC family of cytidine deaminases. The APOBEC family of proteins play important roles in the innate immune response against virus infections by modification of viral genome^11,12^, although they also might serve as endogenous carcinogenic mutagens^13^. These data imply that the overactivity of APOBEC family genes may be induced in response to EBV infection and participate in the tumorigenesis of pulmonary LELC.

To verify the mutations identified in discovery cohort and to better define the mutation patterns of pulmonary LELC, we performed TDS on a panel of 114 selected genes (Supplementary Fig. 7a) for the 29 cases from discovery cohort and a validation cohort of additional 61 cases with a mean coverage of 300× and 170×, respectively (Supplementary Fig. 3b, c and Supplementary Data 3). Cross comparison of somatic mutations in discovery cohort showed that 99% of candidate mutations in WES were confirmed in TDS with a high consistency (0.89) of mutation frequency (Supplementary Fig. 7b and Supplementary Data 5). Combining the data from both cohorts (*n* = 91 subjects), we discovered that 19 genes were affected by non-silent mutations in at least three patients (Fig. 1c and Supplementary Data 4, 6, and 7). Among them, genes with a prevalence of at least 5% included four tumor suppressor genes previously implicated in cancer (*TP53*, *NOTCH1*, *MGA*, and *PTPRD*), one negative regulator of NF-κB pathway (*TRAF3*), one epigenetic modifier (*KMT2C*), and one laminin subunit essential for basement membrane (*LAMA4*). *TP53*, *TRAF3*, *MGA*, *PTPRD*, and *KMT2C* were predicted to be mutually exclusive by MEGSA (corrected *P* *=* 0.005; Supplementary Fig. 8a)^14^, indicating their independent contributions to the carcinogenesis of pulmonary LELC. Although *TP53* mutation was the most frequent in our cohort and twice the frequency of previously reported in pulmonary LELC^15^, it is infrequent as compared to that of other primary lung cancers (Supplementary Fig. 8b). Nevertheless, all the *TP53* mutations were located at the structural domains and 93% (13/14) of these mutations were in the DNA-binding domain (Supplementary Fig. 8c), suggesting the biological consequence of these mutations. Yet, no correlation between *TP53* mutation status and patient’s survival was observed (Fisher’s exact test, *P* = 0.72). Notably, we observed frequent and mutually exclusive mutations of laminin subunit genes (Fig. 1c and Supplementary Fig. 8d), including *LAMA4* (five mutations), *LAMA2* (three), and *LAMB1* (three). The dysregulation of these laminin subunit genes has been widely reported to promote tumor invasion and metastasis in different cancers^16–19^. These imply that the frequent mutation of laminin subunit genes may also play important roles in the progression of pulmonary LELC and warrant further investigation.

Interestingly, frequently altered driver genes (eg. *EGFR*, *KRAS*, and *BRAF*) in other lung cancer subtypes were rarely detected in pulmonary LELC, in consistent with previous reports^6,20–23^. Although *MET* missense mutations were detected in two patients, none of them belong to the canonical *MET* exon 14 skipping mutations^24,25^. These indicate that typical driver mutations in other lung cancer subtypes do not play a critical role in the carcinogenesis of pulmonary LELC.

### Somatic copy number alterations of pulmonary LELC

**Fig. 2:**
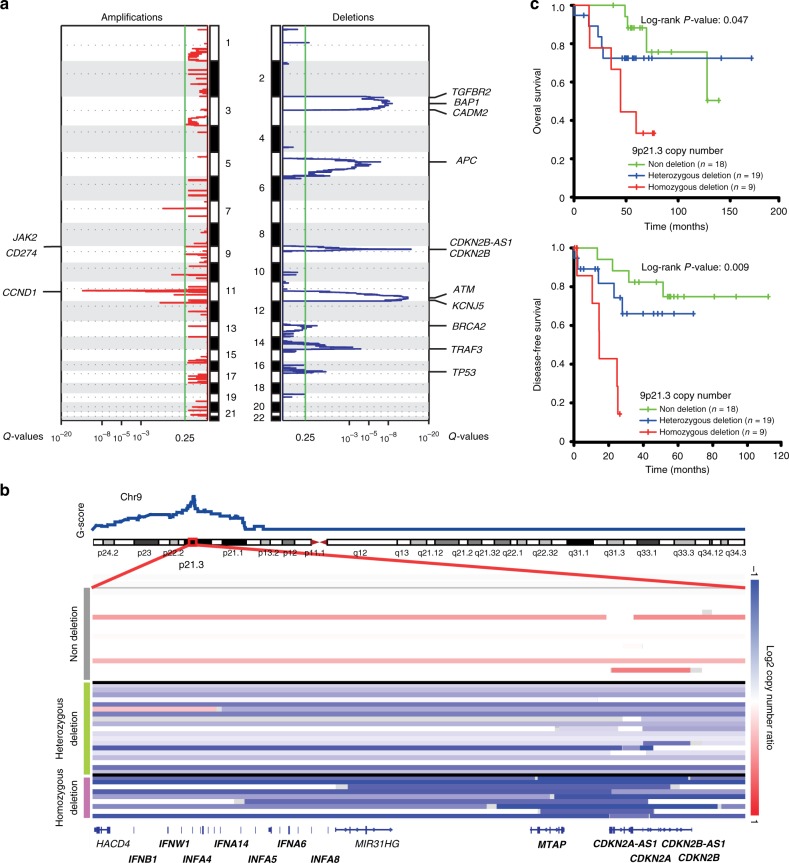
Analysis of copy number alterations in pulmonary LELC. **a** Focal amplification and deletion determined from GISTIC 2.0 analysis. The plot shows significant amplification (red) or deletion (blue) for the chromosomes from 1 (top) to 22 (bottom). The green line indicates the cut-off for significance (*q* = 0.25). Genes listed on left (for amplifications) and right (for deletions) are likely drivers located in the peak areas defined by GISTIC 2.0. **b** Zoom in the significant deletion region in p21.3 of chromosome 9. Samples are classified into three groups: samples without deletions; samples with heterozygous deletions; and samples with homozygous deletions. **c** Kaplan–Meier survival analysis for three groups with different copy number status of 9p21.3. Statistical significance was estimated by two-sided log-rank test. LELC, lymphoepithelioma-like carcinoma

Somatic copy number alterations (SCNAs) were profiled in 46 tumors with sufficient quantity and quality. The number of genes affected ranged from 2381 to 8420 (mean 4600; Fig. 1a and Supplementary Fig. 9a). Frequent arm-level alterations included copy number gains in 5p (32%), 12p (54%), and 12q (48%) and copy number losses in 3p (49%), 5q (47%), 13q (36%), 14q (60%), and 16q (39%) (Supplementary Fig. 9b). Similar to NPC, frequent losses in 14q and 16q were also identified in pulmonary LELC, leading to inactivation of multiple negative regulators of NF-κB pathway (*TRAF3* [14q32.3, 80%], *NFKBIA* [14q13, 52%], *NLRC5* [16q13, 52%], and *CYLD* [16q12.1, 48%])^26,27^. In addition, copy number gain of the whole chromosome 12 was observed in 48% (22/46) of patients, leading to amplification of 34 cancer related genes annotated in the Catalogue of Somatic Mutations in Cancer (COSMIC) database and signaling pathways including MAPK (*q* = 0.001), JAK/STAT (*q* = 0.014), and cell cycle (*q* = 0.032) (Supplementary Data 8). Nineteen significant focal copy number alterations (8 amplifications and 11 deletions) were identified using GISTIC2 (Fig. 2a and Supplementary Data 9)^28^. Significantly amplified regions included 7p11.2, 9p24.1, 11q13.3, and 12p13.2. The predominant event was amplification in 11q13.3, which contained the *CCND1* gene. Amplification of *CCND1* may drive cell cycle progression and contribute to tumorigenesis. Furthermore, we found that *CD274* was amplified in seven cases (8%). Amplification of *CD274* was associated with elevated programmed cell death ligand-1 (PD-L1) expression in EBV-associated gastric cancer^29^. Indeed, we and others have previously found that PD-L1 was remarkably over-expressed in pulmonary LELC^6,23^. The amplification of *CD274* identified herein may provide an alternative mechanism of the overexpression of PD-L1 in pulmonary LELC and the rationale for immunotherapy. Significantly deleted regions included 3p21.31, 3p25.3, 5q14.1, 9p21.3, 11q23.3, 13q14.2, 14q32.32, and 17p13.1 encompassing a great number of tumor suppressor genes such as *BAP1*, *VHL*, *APC*, *ATM*, *RB1*, *TRAF3*, and *TP53* (Fig. 2a and Supplementary Data 9).

Frequent deletion of 9p21.3 was observed in a variety of cancers including NPC and lung cancer^30–32^. In pulmonary LELC, a narrow region (chr9:22028316–22041442) of 9p21.3 was also identified as focal and significant deleted by GISTIC2 (Supplementary Data 9). Patients with 9p21.3 focal deletion were significantly associated with poor survival (Fig. 2c). In addition to the focal deletion region, nearby regions within 9p21.3 also showed high frequency of deletion involving two tumor suppressors (*CDKN2A*, *CDKN2B*), *MTAP* and a cluster of type I IFN genes (Supplementary Data 10, Fig. 2b). The loss of type I IFN genes included all the 13 IFN-α protein coding genes (*IFNA1*, *IFNA2*, *IFNA4*, *IFNA5*, *IFNA6*, *IFNA7*, *IFNA8*, *IFNA10*, *IFNA13*, *IFNA14*, *IFNA16*, *IFNA17*, and *IFNA21*), *IFNE*, *IFNB1* and *IFNW1*, affecting a total of 56% cases (26/46). Biologically, type I IFNs are responsible for the front-line defense against viral infection and are key component in the host-virus standoff^33^. Interestingly, we found that tumors with 9p21.3 deletion had lower level of CD8 + tumor infiltration lymphocytes (TILs) than those without 9p21.3 deletion, with marginal significance (Student’s *t*-test, *P* = 0.05; Supplementary Fig. 10a). It is hypothesized that frequent loss of type I IFN genes may lead to the defect of host immune response against virus and the persistent EBV infection in pulmonary LELC.

### Pathway analysis

**Fig. 3:**
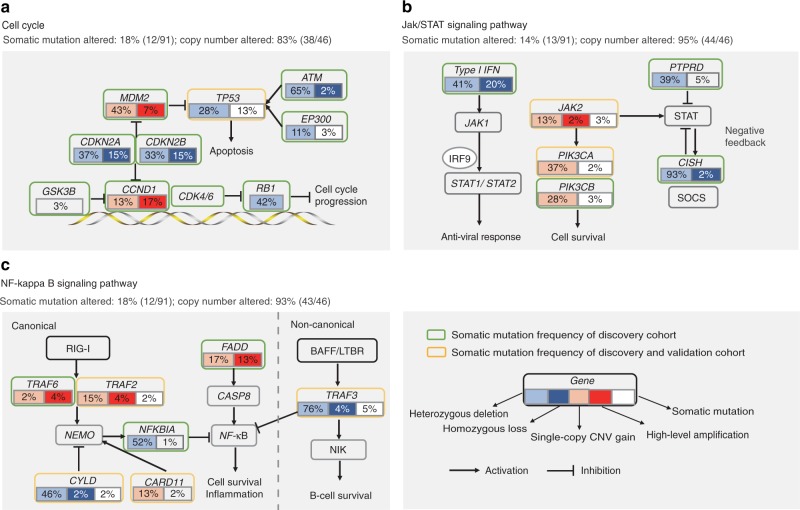
Altered pathways in pulmonary LELC. Alterations defined as somatic mutations, focal amplifications, and deletions affecting Cell cycle (**a**), JAK/STAT (**b**), and NF-kappa B (**c**) signaling pathways are shown. Alteration frequencies are expressed as a percentage of samples form discovery cohort and validation cohort. LELC, lymphoepithelioma-like carcinoma

Integrated analysis of mutational profiles revealed core signaling pathways implicated in discrete functional categories, including cell cycle, JAK/STAT and NF-κB (Fig. 3). Cell cycle pathway was altered primarily by mutation or deletion of *TP53*, amplification of *MDM2* and *CCND1*, and deletion of *CDKN2A*/*B* and *RB1*, revealing frequent defects in the G1/S transition control (Fig. 3a). JAK/STAT pathway was frequently dysregulated, largely owing to deletion of *CISH* (in 95% of the patients), followed by mutation or deletion of *PTPRD*, and mutation or amplification of *JAK2* (Fig. 3b). *CISH* encodes the cytokine-inducible SH2-containing protein from the suppressors of cytokine signaling (SOCS) family which are the major negative regulators of the JAK/STAT pathway^34^. *PTPRD* encodes a tumor suppressor that negatively regulates JAK/STAT pathway by dephosphorylating and inactivating STAT3 oncoprotein^35^. *JAK2* and its downstream signaling cascade genes such as *PI3KCA/B* were also frequently altered in pulmonary LELC. It is of note that JAK/STAT pathway could be activated by IFNs in response to pathogen invasion and induce the transcription of numerous IFN-stimulated genes (ISGs)^36^. Therefore, the widespread deletion of type I interferon genes discussed above could lead to defects in the IFN-induced JAK/STAT activation and the subsequent anti-viral immune response in pulmonary LELC. NF-κB pathway aberration was implicated in 18% of patients by somatic mutations and 93% of patients by SCNAs (Fig. 3c). Negative regulators of NF-κB pathway including *TRAF3*, *CYLD*, *NFKBIA*, and *NLRC5* were frequently deleted in pulmonary LELC. Moreover, recurrent somatic mutations of *TRAF3* and *CYLD* were identified in five and two cases, respectively. Defects in *TRAF3*/*CYLD* have been implicated in the activation of NF-κB signaling in HPV-positive head and neck squamous cell carcinoma (HNSCC) and EBV-positive NPC^26,37^, suggesting that *TRAF3* and *CYLD* genetic alterations may also participate in EBV-mediated tumorigenesis in pulmonary LELC. In addition to deletion of negative regulators, somatic mutations or amplification of multiple components of the canonical NF-κB pathway were also identified, including *FADD*, *TRAF2*, *TRAF6*, and *CARD11*. FADD is an apoptotic adapter molecule that activates the NF-κB pathway by recruiting caspase-8. TRAF2 and TRAF6 are two members of the TNF receptor associated factor (TRAF) protein family, which mediate activation of NF-κB pathway and are involved in the regulation of inflammation, antiviral responses, and apoptosis^38^. *CARD11* encodes the caspase recruitment domain-containing protein 11 that functions as a positive regulator of NF-κB activation by interacting with and inducing phosphorylation of BCL10^39,40^.

### Pathway aberrations are mutually exclusive with LMP1

LMP1 is a viral oncoprotein that potentially activates the NF-kB and JAK/STAT pathways, and promotes cell cycle progression in NPC^41–43^. High LMP1 expression was detected in 19 (20.9%) of 91 pulmonary LELC cases by immunohistochemistry, similar to that in NPC^44^. We also identified mutual exclusivity among LMP1 overexpression and the three core pathway aberrations (Supplementary Data 11) including NF-kB (Fisher’s exact test*, P* *=* 0.00005; Supplementary Fig. 11a), JAK/STAT (Fisher’s exact test*, P* *=* 0.00934; Supplementary Fig. 11b) and cell cycle (Fisher’s exact test*, P* *=* 0.00954; Supplementary Fig. 11c). These data support the hypothesis that somatic genetic alterations and viral-mediated events synergistically participated in the carcinogenesis of pulmonary LELC.

### Immune microenvironment in relation to genomic alterations

Immunostaining for PD-L1 was observed in the membrane and/or cytoplasm of the tumor cells and stromal lymphocytes. We restricted our analysis to CD8 positive TILs due to the fact that these cells are generally thought to be the main effector population following treatment with immune checkpoint inhibitors. Representative PD-L1 and CD8 staining is shown in Supplementary Fig. 1. We found that tumors with 9p24.1 amplification (where *CD247* gene located) was significantly associated with higher PD-L1 expression than those without 9p24.1 amplification (Student’s *t*-test, *P* = 0.01; Supplementary Fig. 10b). However, there were no significant associations between PD-L1 overexpression and number of signature 2 mutations, percentage of EBV type 1 reads, somatic mutation burden or the three core signaling pathways (Supplementary Fig. 10c). Also, no significant correlation was observed between CD8 + TILs and number of signature 2 mutations, percentage of EBV type 1 reads, somatic mutation burden or the three core signaling pathways (Supplementary Fig. 10c).

### TRAF3 is a tumor suppressor in pulmonary LELC

**Fig. 4:**
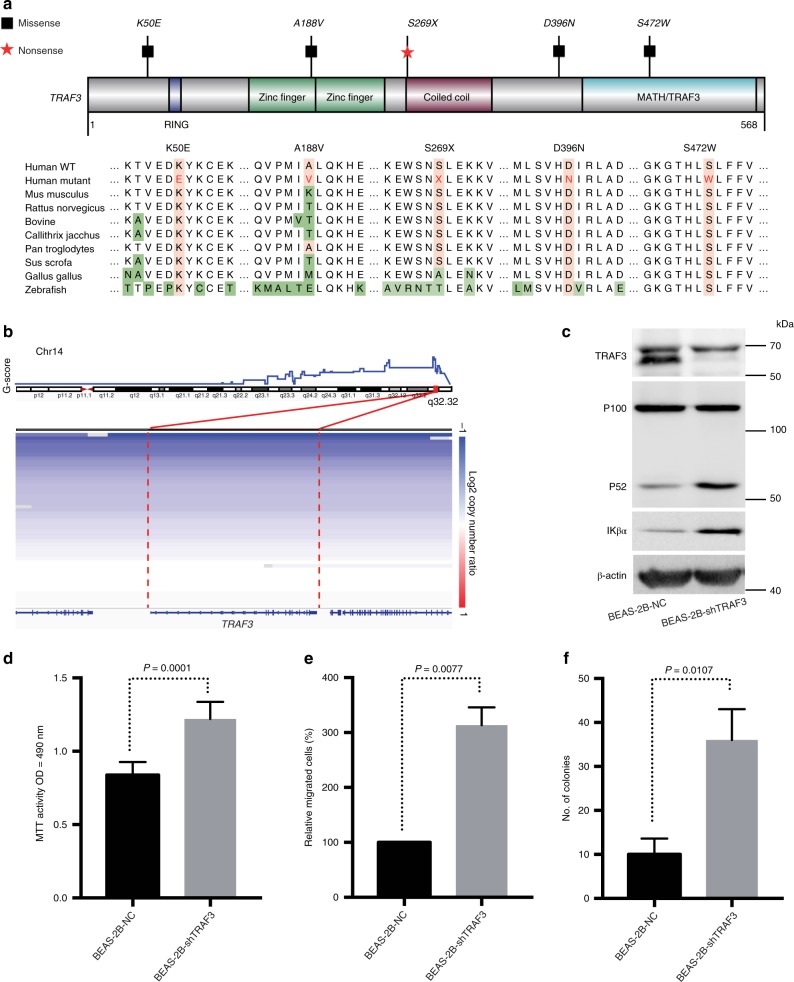
*TRAF3* alterations in pulmonary LELC. **a** Protein domain structure of TRAF3 based on UniProt database with mutated sites. Sequence alignment of TRAF3 protein across distinct species is shown. Amino acid positions of the mutations are indicated above the alignment. **b** Copy number deletions of TRAF3, samples are sorted by log2 copy number ratio. BEAS-2B cells either with or without knockdown of endogenous TRAF3 expression with short hairpin RNAs (shRNAs) were examined by western blot analysis (**c**), MTT assay (**d**), migration assay (**e**), and colony formation assay (**f**). Error bars in **d**, **e**, and **f** denote standard error of the mean. Experiments for **d**, **e**, and **f** were performed in triplicate. Student’s *t*-test was used for statistical analysis of **d**, **e**, and **f**. NC, normal control; MTT, 3-(4, 5-dimethylthiazol-2-yl)-2, 5-diphenyltetrazolium bromide; OD, optical density

TRAF3 functions as a negative regulator of the non-canonical NF-κB pathway^45^. It can also interact with EBV-encoded latent infection membrane protein-1 (LMP1), which may be essential for the oncogenic effects of LMP1 in NPC^41^. In our pulmonary LELC cohort, *TRAF3* mutation was identified in five cases (Fig. 1c and Supplementary Data 4 and 6). Among them, one was nonsense mutation that results in truncated protein product and the other four mutations affected highly conserved residues (Fig. 4a), suggesting that these mutations may alter the protein function and are biologically consequential. Furthermore, *TRAF3* deletion was ubiquitously observed in pulmonary LELC (80%) (Fig. 4b). Given the frequent *TRAF3* aberrations in pulmonary LELC, we further examined its biological function in BEAS-2B cells. Knockdown of wild-type endogenous *TRAF3* expression with short hairpin RNAs (shRNAs) up-regulated key components of NF-κB signaling pathway including p52 and IKβα (Fig. 4c) and led to markedly increased cell growth, cell migration, and colony formation (Fig. 4d–f). These findings indicate that *TRAF3* functions as a tumor suppressor gene and may participate in the tumorigenesis of pulmonary LELC.

### Comparison with other lung cancers and NPC

**Fig. 5:**
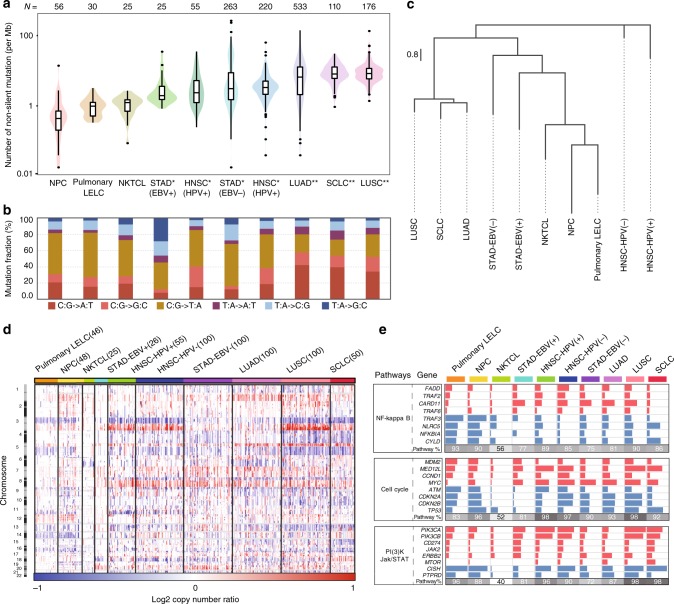
Comparison between pulmonary LELC and other associated cancer types. **a** Distribution of non-silent mutation rates of pulmonary LELC and other associated cancer types. The upper numbers indicate the number of samples for each cancer type. Black lines in the boxplot denote median mutation rate for each cancer type and outliers are shown as dots. All the other cancer types are statistically tested by unpaired two-side *t*-test with pulmonary LELC. *P-value < 0.05 and **P-value < 0.0001. **b** Mutation spectrum of six mutation type for each cancer type. **c** Clustering of 96 subtypes based on six mutation types and nucleotides flanking the mutated base for each cancer type. **d** SCNA comparison of pulmonary LELC and other cancer types. **e** Frequency comparison of genes with copy number amplification (red) or deletion (blue) for three major oncogenic pathways: NF-kappa B, Cell cycle and Jak/STAT/PI(3)K. Percentages of samples mutated in each cancer type are shown in gray. NPC, nasopharyngeal carcinoma; LELC, lymphoepithelioma-like carcinoma; NKTCL, natural killer/T cell lymphoma; STAD, stomach adenocarcinoma; HNSC, head and neck squamous cell carcinoma; EBV, Epstein-Barr virus; HPV, human papillomavirus; LUAD, lung adenocarcinoma; SCLC, small-cell lung carcinoma; LUSC, lung squamous cell carcinoma; SCNA, somatic copy-number alterations; Mb, megabase

Given the ambiguous classification of pulmonary LELC, we comprehensively compared its genetic features with lung adenocarcinoma (LUAD), lung squamous cell carcinoma (LUSC), small-cell-lung carcinoma (SCLC), NPC, natural killer/T-cell lymphoma (NKTCL), EBV-positive and EBV-negative stomach cancer, and HNSCC. Analysis of mutation rate, mutation spectrum of six substitution categories or hierarchical clustering based on 96 trinucleotide mutational contexts demonstrated that pulmonary LELC resembled NPC and NKTCL but was clearly different from other lung cancers (Fig. 5a–c and Supplementary Fig. 12a, b). All lung cancer subtypes except pulmonary LELC were characterized by frequent C:G > A:T transversions of tobacco smoking fingerprint. Moreover, clustering by the mutation frequency of significantly altered genes revealed that other lung cancers had broad spectra of mutated genes, whereas pulmonary LELC, NPC, and NKTCL harbored rare gene mutations (Supplementary Fig. 12c). To avoid the bias caused by the low mutation rate of EBV-positive cancers, we carried out SCNAs comparison (Fig. 5d and Supplementary Fig. 13a). Again, we found pulmonary LELC had very different SCNAs landscape from other lung cancers. LUAD, LUSC, and SCLC showed apparent amplifications of 1q, 5p, 8q and deletion of 8p, which were absent in pulmonary LELC. Notably, NKTCL showed less SCNAs than pulmonary LELC and NPC did and had an evident higher propensity for deletion of 6q, consistent with previous studies^46,47^. Both pulmonary LELC and NPC showed amplification of the whole chromosome 12 and deletion of 3p (contained *BAP1*), 13q (*RB1*), 14q (*TRAF3*), and 16q (*CYLD*). Hierarchical clustering by SCNAs confirmed that pulmonary LELC was grouped with NPC (Supplementary Fig. 13b). Next, we compared the frequency of SCNAs in genes involved in three major pathways (Fig. 5e). We found that pulmonary LELC shared similar altered frequency for almost all the evaluated pathway genes with NPC, particularly NF-κB. Finally, we found similar abundance of EBV sequences between pulmonary LELC and NPC in the WES data, which was much higher than that of NKTCL (Supplementary Fig. 14).

### Gemcitabine has better antitumor activity than pemetrexed

Gemcitabine plus cisplatin has been demonstrated to improve survival of metastatic or recurrent NPC compared to fluorouracil plus cisplatin; while the efficacy of pemetrexed for NPC was very limited^48–50^. As pulmonary LELC resembles NPC in terms of genetic and histopathological features but was currently classified as non-squamous cell lung carcinoma, we retrospectively evaluated the efficacy of gemcitabine plus platinum (GP, *n* = 21) vs. pemetrexed plus platinum (AP, *n* = 38) as first-line treatment for metastatic pulmonary LELC. Among the patients, 27 (46%) were male, 19 (32%) had a history of smoking and the median age at diagnosis was 49 years (range: 29–74). Baseline characteristics of this cohort are presented in Supplementary Data 12. The results showed that GP significantly improved objective response rate (76.19% vs. 23.68%; Pearson’s *χ*^2^ test, *P* *<* 0.001) and progression-free survival (median 8.80 vs. 6.53 months; Log-rank test, *P* *=* 0.009) compared to AP (Supplementary Fig. 15). In multivariate analysis controlling for potential confounding factors including age, gender, performance status, stage, and number of metastatic organs, gemcitabine plus cisplatin remained significantly associated with improved progression-free survival (Cox proportional-hazards regression, *P* = 0.024) and overall response (Cox proportional-hazards regression, *P* = 0.001) compared with pemetrexed plus cisplatin (Supplementary Data 13).

## Discussion

In summary, our study of 91 pulmonary LELCs using WES, TDS, SNP arrays analysis and functional experiments revealed the distinct mutational landscape of this special subtype of lung cancer. We identified an infrequent somatic mutation rate but the widespread existence of copy number alterations in pulmonary LELC. We also discovered novel genomic events affecting several key pathways that might contribute to the tumorigenesis of this disease, capital among these being cell cycle, JAK/STAT and NF-κB. The involvement of viral infection in pulmonary LELC pathogenesis was also demonstrated in the study. More importantly, by multidimensional genomic comparison, we unveiled that pulmonary LELC is a unique subtype of lung cancer that genetically resembles NPC.

Given the rarity of somatic driver mutations in pulmonary LELC and the fact that this disease is closely related to EBV infection, the underlying mechanism of tumorigenesis is of special interest. In this study, we detected positive EBV-encoded RNA (EBER) staining by in situ hybridization (ISH) and EBV sequence by WES for all the tumor cases, confirming the presence of EBV infection in pulmonary LELC. Besides, we identified widespread signature 2 mutations, which are attributed to the overactivity of AID/APOBEC family of cytidine deaminases that participate in the antiviral innate immune response partially through inducing transcription of type I interferons, as well as modification of viral genome. However, accumulating evidence also suggests that APOBEC family proteins could on the other hand serve as endogenous mutagens for carcinogenesis. Furthermore, the frequently dysregulated NF-κB pathway could be hijacked by the invading viruses to prolong survival of the host cell in order to buy time for viral replication and progeny production^51^. Notably, we also identified ubiquitous losses of type I IFN genes in pulmonary LELC, which led to defect in the production of anti-viral cytokines and IFN-dependent JAK/STAT activation. Previous studies showed that type I interferon might enhance CD8 + T cell effector function, systematically activate natural killer (NK) cell activity and increase antigen presentation of the tumor cells to be recognized by T lymphocytes^52–54^. Therefore, it might be inferred that the frequent loss of type I interferon genes may impair the efficacy of immune checkpoint inhibitor therapy. Collectively, the APOBEC family gene signature, dysregulated NF‐κB pathway and loss of type I IFN genes are likely responsible for the EBV-induced carcinogenesis of pulmonary LELC and might facilitate development of novel therapeutic strategies.

We also found that *TRAF3* was ubiquitously altered in pulmonary LELC, including 5% of simple somatic mutation and 80% of deletion. Functional experiments confirmed that *TRAF3* served as a tumor suppressor gene and negatively regulated NF-κB pathway. Therefore, *TRAF3* loss accounts for the core element of NF-κB dysregulation and plays important role in tumorigenesis of pulmonary LELC. It could be conceived of that NF-kB inhibitors can potentially be used as novel therapeutics in pulmonary LELC patients.

Finally, besides the known histological similarity with NPC, we provided the first comprehensive genetic landscape comparison between pulmonary LELC and NPC, as well as other primary lung cancers. We revealed clear difference of mutation spectrum, significant somatic mutations, copy number alterations, and signaling pathway aberrations between pulmonary LELC and other lung cancers; whereas, pulmonary LELC resembles NPC genetically, e.g., low degree of somatic mutation, exclusivity of LMP1 overexpression with somatically altered signaling pathways, frequent chromosomal alterations and predominant NF-κB dysregulation. In addition, we showed that gemcitabine might improve response rate and progression-free survival compared with pemetrexed as first-line palliative chemotherapy in metastatic pulmonary LELC. This preliminary clinical data may also add evidence for the similarity between this unique lung cancer and NPC.

One major limitation of the current study is that the methods we applied could not provide other genomic information such as chromosomal alterations and transcriptome. Further studies are needed to fully unveil the genomic architecture of pulmonary LELC. Secondary, because this study focused on the genomic features of this special tumor, we did not dig into the exact viral integration sites or the detailed mechanisms of EBV-induced carcinogenesis. However, our study did have provided important insights into further research in this area.

In conclusion, our study delineated a comprehensive view of genomic alterations in pulmonary LELC, defined potential mechanism of tumorigenesis and provided evidence that pulmonary LELC is a distinct lung cancer that resembles NPC. The data presented here might offer novel avenues for treatment of this lethal malignancy and are important for future revision of histological classification of lung tumors.

## Methods

### Patients and samples

The study retrospectively collected fresh-frozen tumor tissue and matched tumor adjacent normal tissue as well as Formalin-fixed, Paraffin-embedded (FFPE) tissue from 91 pulmonary LELC patients for genomic characterization. All the patients had surgical resection in Sun Yat-sen University Cancer Center (SYSUCC) between April 2002 and April 2014 (Supplementary Data 1 and 2). An additional cohort of 59 metastatic pulmonary LELC who received first-line palliative chemotherapy between April 2011 and September 2017 were retrospectively included for further survival analysis (Supplementary Data 12). The primary endpoints of the second cohort analysis were objective response rate and progression-free survival between two treatment groups. Nasopharyngoscopy or Magnetic Resonance Imaging (MRI) was done to rule out lung metastasis from NPC in all the patients. Pathological diagnoses were established according to the WHO classification and independently reviewed by two pathologists. All tumor cases were confirmed to be positive for EBV-encoded RNA (EBER) staining determined using the EBV Probe In Situ Hybridization (ISH) Kit (Triplex International Biosciences, China). For the genomic study, proportion of tumor content must be 30% or more (Supplementary Data 1). Detailed clinical characteristics were summarized in Supplementary Data 1, 2, and 12. The study protocol was approved by the Institutional Review Board of SYSUCC (B2015-005-01). All the patients have provided written informed consent.

### DNA extraction

For fresh-frozen samples (discovery cohort), genomic DNA from tumors and matched normal samples were isolated using the QIAamp DNA Mini Kit (Qiagen), according to the manufacturer’s instructions. And for FFPE tumors (validation cohort), DNA was extracted using QIAamp DNA FFPE Tissue Kit (Qiagen). All DNA was quantified using the Qubit Fluorometer, and the quality of DNA was tested using agarose gel electrophoresis.

### Whole-exome sequencing

To construct whole-exome capture libraries, 2 μg of genomic DNA form each fresh-frozen tumor and matched normal sample was randomly fragmented by Covaris into 200~250 bp. After fragmentation, these fragments were purified and ligated by BGI-designed PE Index Adaptors, then captured with the BGI-Exome-V4 kit (~59 Mb; BGI, Shenzhen, China). All the constructed libraries were loaded on Hiseq4000 platform (Illumina) and the sequences were generated as 150-bp paired-end reads.

Sequencing reads which contained sequencing adapters, more than 10% of unknown bases and low-quality bases (>50% bases with base quality <5) were removed. Processed sequencing reads were then aligned to UCSC human reference genome (hg19) using BWA-MEM (v0.7.12). Picard (v1.84, http://broadinstitute.github.io/picard/) was used to generate chromosomal coordinate-sorted bam files and to remove PCR duplications. Then we performed base quality score recalibration and local realignment of the aligned reads using the Genome Analysis Toolkit (GATK v3.4)^55^ to improve alignment accuracy.

### Somatic mutation detection

After sequencing data processed, the potential somatic substitutions (SNVs) were called by MuTect (v1.1.7)^56^ with default parameter based on paired alignment files (tumor and matched normal). Somatic InDels were predicted with GATK SomaticIndelDetector with default parameters. In this process, the following SNVs were eliminated: (i) mutations reported in dbSNP (v142) with mutated allele supporting reads <10; (ii) distance between two mutations is <3; (iii) strand bias (either plus or minus strand supporting reads divided by total variant supporting reads) is >0.9; (iv) mutations reported in 1000 Genome Project April 2015 release; the National Heart, Lung, and Blood Institute (NHLBI) Grand Opportunity (GO) Exome Sequencing Project (ESP) ESP6500SI-V2 release and The Exome Aggregation Consortium (ExAC) database release 0.3 with a frequency of >0.01. For somatic InDels, mutations were further removed if the supporting reads from both tumor and normal samples was <5, median/mad of InDel offsets from the starts or ends of the reads ≥5 bp, average mapping qualities of the reads supported reference and InDel in tumor samples was ≤20, or in simple repeat regions. All SNVs and InDels were subsequently annotated by ANNOVAR.

### Targeted sequencing

To determine the mutations of candidate genes, we performed target deep sequencing on 114 carefully selected genes (Supplementary Fig. 7a). Genes were selected with the following criteria:

- Genes mutated in at least two of the 30 WES patients. These genes will be manually checked with literatures by two researchers in our institute to ensure the association with cancer. In addition, we also included genes mutated in one patient only if it was presented in the cancer gene census (CGC) database of COSMIC (https://cancer.sanger.ac.uk/census).
- Genes frequently mutated in NPC^30^, NKTCL^47^, LUAD (TCGA)^31^, LUSC (TCGA)^32^, and SCLC^57^. In addition, the top 20 frequently mutated genes provided by the Cancer Browse tools in COSMIC database (https://cancer.sanger.ac.uk/cosmic) for LUSC, LUAD, and SCLC were also considered. These genes were also manually checked as described in a).
- We also included NF-kB pathway genes that mutated in only one out of the 30 WES patients or frequently mutated in other cancers.

We then merged and removed duplicates for all genes. Thus, we have three categories of unique genes included in the 114 gene panel: cancer associated genes frequently mutated in pulmonary LELC or other related cancers, and NF-kB pathway genes. A list of genes for each category was shown in Supplementary Fig. 7a. And a customized DNA enrichment kit, capturing all exons from these 114 genes and targeting ~700 k genomic regions, was designed. Genomic DNA (200–500 ng) from each sample for validation was used for hybrid capture and library construction. Libraries were then sequenced on Hiseq4000 platform (Illumina) with 2 × 150 bp paired-end reads. Sequenced reads were processed as WES data described above.

We validated the somatic mutations identified in WES by observing at least three reads supporting the mutant allele in the target deep sequencing. In addition, the Pearson correlation coefficient was calculated to estimate the consistency of mutation frequency identified in WES and TDS.

An in-house pipeline was employed to detect SNVs for FFPE samples that do not have matched normal controls. First, we examined all candidate mutation sites by excluding those: (i) alignment quality <20 and sequencing base quality <20; (ii) sequencing depth <10 and mutant allele supporting reads <4; (iii) mutation frequency <5% or between 45 and 55%; (iv) registered in dbSNP142, ExAC, ESP6500, and the 1000 Genomes project with a frequency of >0.01 unless they were registered in the COSMIC; (v) presented in our SNP database constructed from the 30 normal samples in discovery cohort; (vi) distance between two mutations <3 or strand bias >0.9. We tested the pipeline on 30 tumors which had both WES and TDS data and achieved an overall validation rate of 82%.

### Mutational signature analysis

Mutational signature was first identified using the BayesNMF algorithms. Firstly, the count of somatic mutations was calculated for each type of substitution (96 trinucleotide mutation contexts) to generate the mutational catalogue. Then we ran the Bayesian NMF 1000 times with the hyperparameter for the inverse gamma prior setting to 10 (*a* = 10); the iterations were terminated when the tolerance for convergence was <10–7; and half-normal was chose as ‘pirors’ for this algorithm. We identified two significant signatures in our data set. To increase the confidence of the findings, we also used the NMF methodology described by Alexandrov et al.^10^ (http://www.mathworks.com/matlabcentral/fileexchange/38724). The number of cycles of NMF runs was set from 1 to 15, and 2 was the best estimation due to the high stability and low reconstruction error. The two signatures were then compared to the known mutational processes from the COSMIC signature database (http://cancer.sanger.ac.uk/cosmic/signatures) by calculating the cosine similarity as following:^58^

$${\mathrm{sim}}\left( {A,B} \right) = \frac{{\mathop {\sum }\nolimits_{K = 1}^K A_KB_K}}{{\sqrt {\mathop {\sum }\nolimits_{K = 1}^K (A_K)^2} \sqrt {\mathop {\sum }\nolimits_{K = 1}^K (B_K)^2} }}$$

where *K* is the number of mutation types (*K* = 96). Because the elements of *A* and *B* are nonnegative, the cosine similarity has a range between 0 and 1. When the cosine similarity is 1 between two signatures, these signatures are exactly the same. In contrast, when the similarity is 0, the signatures are independent.

### Copy number analysis

Analysis of copy number alterations was performed on the basis of DNA profiling of each tumor or normal sample on Affymetrix OncoScan® CNV FFPE Assay. Genomic DNA was quantified using a Qubit™ Fluorometer and at least 80 ng of genomic DNA with DNA concentration ≥12 ng/μl was required for each sample. Before processing according to the OncoScan® CNV FFPE Assay Kit protocol, DNA integrity was verified by agarose gel electrophoresis. The raw intensity data (DAT) were analyzed with Affymetrix® GeneChip® Command Console® (AGCC) software (v4.1.2; Affymetrix) and generated array fluorescence intensity (CEL) files. For FFPE and frozen samples, normalized log R ratio (LRR) and B allele frequency (BAF) for all the available probes in each sample were extracted by the OncoScan Console (v1.2; Affymetrix) from fluorescence intensity (CEL) files using FFPE Analysis NA33 and REF103 Analysis NA33 as normal reference panel, respectively. Samples were excluded from downstream analysis if single-nucleotide polymorphism quality control (SNPQC) ≤ 20 or the median absolute value pairwise difference (MAPD) ≥ 0.3. Segments were then detected by Nexus Express software using the TuScan segmentation algorithm with default parameters. We removed segments that spanned <100 kb or contained <25 probes. Broad and focal CNVs were identified using GISTIC2.0 algorithm^28^ with parameters: -genegistic 1 -broad 1 -brlen 0.98 -conf 0.95 -armpeel 1 -js 8. We also removed regions corresponding to germline copy-number alterations by applying filters generated from the TCGA and our normal samples when performing GISTIC analysis.

### Pathway enrichment analysis

We performed pathway enrichment analysis by integrating somatic mutation and SCNA data using both Gene Set Enrichment Analysis (GSEA, http://software.broadinstitute.org/gsea) and DAVID (https://david-d.ncifcrf.gov/). GSEA pathway analysis was based on the Kyoto Encyclopedia of Genes and Genomes (KEGG) database from MSigDB version 6.0. The significance enrichment pathways were determined by a hypergeometric test and the FDR *q*-values was <0.05.

### Comparison analysis

We downloaded the SNV/InDels and CNV data of LUAD, LUSC, STAD, HNSC from The Cancer Genome Atlas (TCGA, http://gdac.broadinstitute.org/). Mutation data of NPC (Lin et al.)^30^, NKTCL (Jiang et al.)^47^, SCLC (George et al.)^57^ were adopted from the latest publications.

Total somatic mutation rate for each cancer type was calculated with the non-silent mutations and tested by Student’s *t*-test. The association between pulmonary LELC and other cancer types of six base substitutions was calculated by Pearson correlation coefficient. Hierarchical clustering of 96 possible mutation types (Six base substitutions each with 16 possible combinations of neighboring bases) was performed by the ‘aheatmap’ function with euclidean distance and ‘ward.D2’ agglomeration method of R package NMF (https://cran.r-project.org/web/packages/NMF/index.html). We also generated a phylogenetic tree by first computing the Pearson correlation between all cancers and using these dissimilarity values to cluster the cancers.

Broad and focal SCNAs were analyzed with GISTIC 2.0 algorithm for each cancer type. The thresholds for gene copy number alterations were: amplifications, GISTIC score = 2; gains, GISTIC score = 1; losses, GISTIC score = −1; deletions, = −2. The landscape and frequency of copy number alterations was displayed on The Integrative Genomics Viewer (IGV, v 2.2.7).

For EBV abundance analysis, sequencing reads that could not mapped to the human reference genome(hg19) were extracted and realigned to the EBV genome type 1 (NC_007605.1) by BWA-MEM (v0.7.12). Then the number of mapped reads was calculated to estimate the EBV abundance among all the whole-exome-sequenced samples.

### Immunohistochemical staining

The expression of LMP1, PD-L1, and CD8 was determined in FFPE pulmonary LELC sections by immunohistochemical staining. After de-waxing, the sections were subjected to antigen retrieval and staining in the automated slide processing system BenchMark XT (Ventana Medical systems Inc., Tucson, AZ) with the OptiView Amplification kit (Ventana Medical Systems Inc.). The primary antibody used in this study was anti-LMP1 mouse monoclonal antibody (CS.1–4, Dako), anti-PD-L1 rabbit monoclonal antibody (E1L3N, Cell Signaling Technology) and anti-CD8 mouse monoclonal antibody (4B11, Leica Microsystems). The LMP1 and PD-L1 expression was assessed by two independent pathologists by assigning a proportion score and an intensity score (0, absent; 1, weak; 2, moderate; and 3, strong). The H-score was the product of proportion multiplied by intensity scores, ranging from 0 to 300. According to the report by Yvonne Y. Li et al., the LMP1 expression was categorized into absence/low (score 0–100) and high (score 101–300)^26^. According to International TILs Working Group 2014, we scored CD8 + TILs as a percentage of positive staining in the stromal areas alone, with areas occupied by carcinoma cells excluded^59^.

### Cell culture

Immortalized human lung bronchial epithelial cell line (Beas-2B) were generously provided by Prof. Liang Chen (Jinan University, Guangzhou, China)^60^. All the cell lines were cultured in DMEM supplemented with 10% fetal bovine serum and antibiotics (10,000 U/mL penicillin and 10 mg/mL streptomycin). All the cells were maintained in a humidified incubator at 37 °C with 5% CO_2_.

### Knockdown of endogenous TRAF3

The sequences of the *TRAF3* siRNA were designed by RIBOBIO (Guangzhou, China) as following: sense, (5′−3′) 5′-GGAAGAUUCGCGACUACAAdTdT-3′ and antisense, 3′-dTdTCCUUCUAAGCGCUGAUGUU-5′. Recombinant lentivirus expressing either vector (GV248) or GV248 subcloned with *TRAF3* siRNA was constructed by GENE Corporation (Shanghai, China). According to the manufacturer’s instructions, lenti-shTRAF3 (shTRAF3) and negative control (shNC) with package vectors were transfected into HEK-293 T cells for 72 h. Lentivirus supernatants were harvested and used to infect BEAS-2B cells with 2 μg/ml polybrene for 48 h. The cells were cultured with 2 μg/ml puromycin (Thermofish Scientific) in the medium for 7 days to construct TRAF3 down-regulated cells (BEAS-2B- shTRAF3), as well as negative control cells BEAS-2B- shNC.

### Migration assay

Beas-2B cells were seeded onto transwell inserts (Corning, 3422) in 24-well plates and incubated for 48 h. The inserts were washed with PBS, and non-migrating cells were wiped off from the top side. Migrated cells were fixed with 4% paraformaldehyde and stained with 0.1% crystal violet solution, and nuclei were counted.

### Colony formation assay

Beas-2B cells were plated on 6-well plates with a density of 300 cells per well in triplicate. After 2 weeks, the cells were fixed with 4% polyformaldehyde, and then stained with freshly prepared diluted 0.1% crystal violet solution for 20 min. After rinsing with distilled water, colonies of 50 or more cells were counted under a stereomicroscope.

### Short-term cell proliferation assays

Beas-2B cells were seeded on the 96-well plates at optimized confluence in triplicate and were grown for a total of 4 days. 3-(4, 5-dimethylthiazol-2-yl)−2, 5-diphenyltetrazolium bromide (MTT) incorporation was performed to quantify the number of cells according to the manufacturer’s guidance (Thermofish Scientific).

### Western blotting

The whole-cell lysates were prepared with extraction buffer (50 mM Tris-HCl, pH 7.4, 150 mM NaCl and 0.5% Nonidet P-40) supplemented with complete protease and phosphatase inhibitor cocktail (Roche). The procedures for standard western blotting were performed according to the manufacturer’s guidance. The antibodies specific for TRAF3 (Cat#: 4729, 1:1000 dilution), P100 (Cat#, 4882, 1:1000 dilution), P52 (Cat#, 4882, 1:1000 dilution), IKβα (Cat#, 2859, 1:1000 dilution), and β-actin (Cat#, 4970, 1:1000 dilution) were purchased from Cell Signaling Technologies, USA. The uncropped and unprocessed scans of Fig. 4c are shown in Supplementary Fig. 16.

### Statistical analysis

Two-tailed Student’s *t*-test and Fisher’s exact test were used for continuous and discrete variables, respectively. Pearson’s chi-squared test was used for comparison of response rate difference. And survival probability and difference were analyzed using log-rank test and a Cox proportional hazards model (multi-variate analysis). All statistical analysis was done with standard R packages. A two-sided *p*-value of <0.05 defined statistical significance.

### Reporting summary

Further information on research design is available in the Nature Research Reporting Summary linked to this article.

## Supplementary information

Reporting Summary

Supplementary Information

Description of Additional Supplementary Information

Supplementary Data 1

Supplementary Data 2

Supplementary Data 3

Supplementary Data 4

Supplementary Data 5

Supplementary Data 6

Supplementary Data 7

Supplementary Data 8

Supplementary Data 9

Supplementary Data 10

Supplementary Data 11

Supplementary Data 12

Supplementary Data 13

## Data Availability

Patient clinical data (deidentified) were provided in the Supplementary Data 1 and 12. The complete somatic mutation calls can be found in Supplementary Data 4, 5 and 6. The VCF of exome sequencing and targeted sequencing that support this study have been deposited both in the European Variation Archive (EVA) at the EMBL-EBI under accession number PRJEB32689 and the CNGB (China National GeneBank) Nucleotide Sequence Archive (CNSA) database under the accession codes CNP0000327 (https://db.cngb.org/cnsa/). All the other data supporting the findings of this study are available within the article and its Supplementary Information Files and from the corresponding authors upon reasonable request.
