# Supplementary material for: The genomic landscape of Epstein-Barr virus-associated pulmonary lymphoepithelioma-like carcinoma
Source: Nat Commun. 2019 Jul 16;10:3108. doi: 10.1038/s41467-019-10902-w (PMC6635366; doi:10.1038/s41467-019-10902-w)
Supplement: Supplementary file 1 — Reporting Summary [file 41467_2019_10902_MOESM1_ESM.pdf]

nature research

Corresponding author(s): Li Zhang and Kui Wu

Last updated by author(s): May 22, 2019

Reporting Summary

Nature Research wishes to improve the reproducibility of the work that we publish. This form provides structure for consistency and transparency in reporting. For further information on Nature Research policies, see Authors & Referees and the Editorial Policy Checklist.

Statistics

For all statistical analyses, confirm that the following items are present in the figure legend, table legend, main text, or Methods section.

n/a	Confirmed	
☐	☒	The exact sample size (*n*) for each experimental group/condition, given as a discrete number and unit of measurement
☐	☒	A statement on whether measurements were taken from distinct samples or whether the same sample was measured repeatedly
☐	☒	The statistical test(s) used AND whether they are one- or two-sided<br>*Only common tests should be described solely by name; describe more complex techniques in the Methods section.*
☐	☒	A description of all covariates tested
☐	☒	A description of any assumptions or corrections, such as tests of normality and adjustment for multiple comparisons
☐	☒	A full description of the statistical parameters including central tendency (e.g. means) or other basic estimates (e.g. regression coefficient) AND variation (e.g. standard deviation) or associated estimates of uncertainty (e.g. confidence intervals)
☒	☐	For null hypothesis testing, the test statistic (e.g. *F*, *t*, *r*) with confidence intervals, effect sizes, degrees of freedom and *P* value noted<br>*Give P values as exact values whenever suitable.*
☒	☐	For Bayesian analysis, information on the choice of priors and Markov chain Monte Carlo settings
☒	☐	For hierarchical and complex designs, identification of the appropriate level for tests and full reporting of outcomes
☒	☐	Estimates of effect sizes (e.g. Cohen's *d*, Pearson's *r*), indicating how they were calculated

Our web collection on statistics for biologists contains articles on many of the points above.

Software and code

Policy information about availability of computer code

Data collection	no software was used.
Data analysis	no software was used.

For manuscripts utilizing custom algorithms or software that are central to the research but not yet described in published literature, software must be made available to editors/reviewers. We strongly encourage code deposition in a community repository (e.g. GitHub). See the Nature Research guidelines for submitting code & software for further information.

Data

Policy information about availability of data

All manuscripts must include a data availability statement. This statement should provide the following information, where applicable:

- Accession codes, unique identifiers, or web links for publicly available datasets
- A list of figures that have associated raw data
- A description of any restrictions on data availability

Patient clinical data (deidentified) were provided in the Supplementary Data 1 and 12. The complete somatic mutation calls can be found in Supplementary Data 4, 5 and 6. The VCF of exome sequencing and targeted sequencing that support this study have been deposited in the European Variation Archive (EVA) at the EMBL-EBI under accession number PRJEB32689 (https://www.ebi.ac.uk/eva/?eva-study= PRJEB32689). All the other data supporting the findings of this study are available within the article and its supplementary information files and from the corresponding authors upon reasonable request.

Field-specific reporting

Please select the one below that is the best fit for your research. If you are not sure, read the appropriate sections before making your selection.

☒ Life sciences ☐ Behavioural & social sciences ☐ Ecological, evolutionary & environmental sciences

For a reference copy of the document with all sections, see nature.com/documents/nr-reporting-summary-flat.pdf

Life sciences study design

All studies must disclose on these points even when the disclosure is negative.

Sample size	No sample size was calculated because pulmonary LELC is a rare subtype of primary lung cancer. Therefore, we collected all the eligible cases from our institute as many as possible. The sample is powered to detect the difference between the groups as illustrated in the final section of the Results.
Data exclusions	No data exclusions.
Replication	To verify the mutations identified in discovery cohort by whole exon sequencing (WES) and to better define the mutation patterns of pulmonary LELC, we performed targeted deep sequencing (TDS) on a panel of 114 selected genes. Cross comparison of somatic mutations in discovery cohort showed that 99% of candidate mutations in WES were confirmed in TDS with a high consistency (0.89) of mutation frequency. Cellular experiments were repeated three times. We confirmed all attempts at replication were successful.
Randomization	Randomization and Blinding were not used in this study, because the purpose of the real-world clinical study was to compare the anti-tumor activity of gemcitabine and pemetrexed as first-line treatment for metastatic pulmonary LELC. These patients was recruited retrospectively. And clinical data were retrieved from digital medial records.
Blinding	Randomization and Blinding were not used in this study, because the purpose of the real-world clinical study was to compare the anti-tumor activity of gemcitabine and pemetrexed as first-line treatment for metastatic pulmonary LELC. These patients was recruited retrospectively. And clinical data were retrieved from digital medial records.

Reporting for specific materials, systems and methods

We require information from authors about some types of materials, experimental systems and methods used in many studies. Here, indicate whether each material, system or method listed is relevant to your study. If you are not sure if a list item applies to your research, read the appropriate section before selecting a response.

Materials & experimental systems

n/a	Involved in the study	
☐	☒	Antibodies
☐	☒	Eukaryotic cell lines
☒	☐	Palaeontology
☒	☐	Animals and other organisms
☐	☒	Human research participants
☐	☒	Clinical data

Methods

n/a	Involved in the study	
☒	☐	ChIP-seq
☒	☐	Flow cytometry
☒	☐	MRI-based neuroimaging

Antibodies

Antibodies used	anti-LMP1 mouse monoclonal antibody (CS.1-4, Dako), anti-PD-L1 rabbit monoclonal anti-body (E1L3N, Cell Signaling Technology), anti-CD8 mouse monoclonal antibody (4B11, Leica Microsystems), the anti-body specific for TRAF3 (#4729), P100 (#4882), P52 (#4882), IKβα (#2859) and β-actin (#4970) was purchased from Cell Signaling Technologies, USA.
Validation	Documents certify that the products have met the quality control standards defined by the manufacturers.

Eukaryotic cell lines

Policy information about cell lines

Cell line source(s)	Cell line sources are listed in page 32.
Authentication	The lines were authenticated by short tandem repeat (STR) profiling.
Mycoplasma contamination	The cell lines were not tested for mycoplasma contamination.

Commonly misidentified lines (See ICLAC register)	no

Human research participants

Policy information about studies involving human research participants

Population characteristics	1. A cohort of 91 pathologically confirmed pulmonary LELC patients (Supplementary Fig.1, 2a, Supplementary Data 1, and Supplementary Data 2) from Sun Yat-sen University Cancer Center, China, provided tumor samples for genomic analysis. Among the patients, 43 (47%) were male, 17 (19%) had a history of smoking and the median age at diagnosis was 53 years (range: 27-71).<br>2. An additional cohort of 59 metastatic pulmonary LELC who received first-line palliative chemotherapy were included from Sun Yat-sen University Cancer Center, China, to compare the anti-tumor activity of gemcitabine vs pemetrexed. Detailed baseline characteristics of this cohort are presented in Supplementary Data 12. Among the patients, 27 (46%) were male, 19 (32%) had a history of smoking and the median age at diagnosis was 49 years (range: 29-74).
Recruitment	The study retrospectively collected fresh frozen tumor tissue and matched tumor adjacent normal tissue as well as Formalin-fixed, Paraffin-embedded tissue from 91 pulmonary LELC patients for genomic characterization. All the patients had surgical resection in Sun Yat-sen University Cancer Center (SYSUCC) between April 2002 and April 2014 (Supplementary Data 1, Supplementary Data 2). An additional cohort of 59 metastatic pulmonary LELC who received first-line palliative chemotherapy between April 2011 and September 2017 were included for further survival analysis (Supplementary Data 12).
Ethics oversight	The study was approved by the Institutional Review Board of Sun Yat-Sen University Cancer Center (B2015-005-01).

Note that full information on the approval of the study protocol must also be provided in the manuscript.

Clinical data

Policy information about clinical studies

All manuscripts should comply with the ICMJE guidelines for publication of clinical research and a completed CONSORT checklist must be included with all submissions.

Clinical trial registration	The clinical data in this study was retrospectively collected rather than being collected from any prospective clinical trial. Therefore, clinical trial registration is not obligatory. The study protocol was approved by the Institutional Review Board of Sun Yat-sen University Cancer Center (B2015-005-01).
Study protocol	Study protocol was deposited in the Research Data Deposit (RDD) of Sun Yat-sen University Cancer Center and could be assessed from the corresponding authors.
Data collection	The study retrospectively collected fresh frozen tumor tissue and matched tumor adjacent normal tissue as well as Formalin-fixed, Paraffin-embedded tissue from 91 pulmonary LELC patients for genomic characterization. All the patients had surgical resection in Sun Yat-sen University Cancer Center (SYSUCC) between April 2002 and April 2014 (Supplementary Data 1, Supplementary Data 2). An additional cohort of 59 metastatic pulmonary LELC who received first-line palliative chemotherapy between April 2011 and September 2017 were included for further survival analysis (Supplementary Data 12). Clinical data were retrieved from digital medical records.
Outcomes	The first cohort of 91 pulmonary LELC patients provided tumor samples for genomic characterization. The second cohort of 59 metastatic pulmonary LELC patients were recruited for the analysis of the anti-tumor activity of gemcitabine vs. pemetrexed as first-line treatment, with pre-defined primary outcomes of objective response rate (ORR) and progression-free survival (PFS).
