# Supplementary material for: The genomic landscape of Epstein-Barr virus-associated pulmonary lymphoepithelioma-like carcinoma
Source: Nat Commun. 2019 Jul 16;10:3108. doi: 10.1038/s41467-019-10902-w (PMC6635366; doi:10.1038/s41467-019-10902-w)
Supplement: Supplementary file 2 — Supplementary Information [file 41467_2019_10902_MOESM2_ESM.pdf]

Supplementary Information

Hong et al.

The Genomic Landscape of Epstein-Barr virus (EBV)-associated pulmonary lymphoepithelioma-like carcinoma

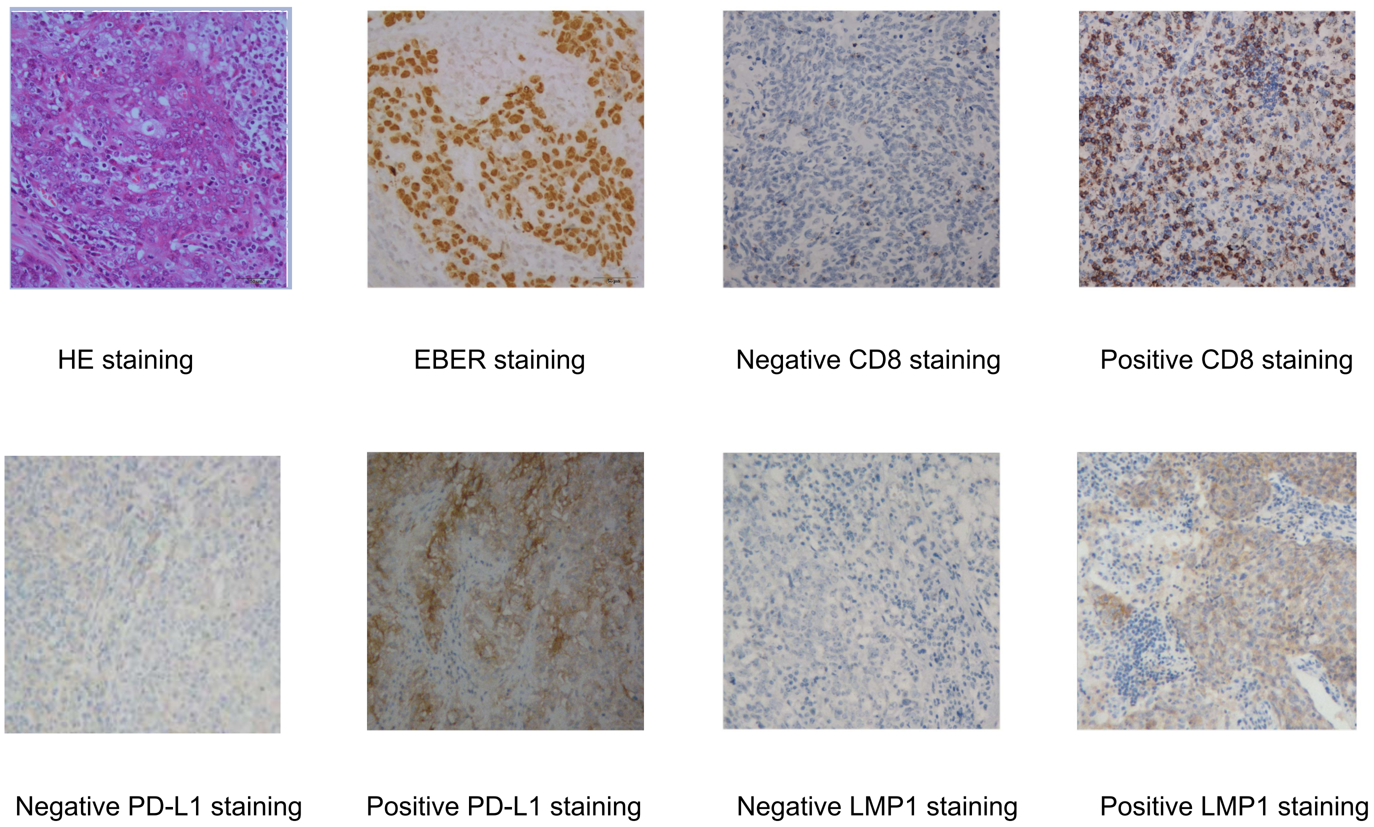

Supplementary Figure 1. Representatives of pathological and immunohistochemical features of pulmonary lymphoepithelioma-like carcinoma. Tumor samples with ≥ 30 tumor content were selected for the study. HE, Hematoxylin and Eosin; EBER, Epstein–Barr virus-encoded small RNA; PD-L1, programmed death 1 ligand 1; LMP1, Epstein–Barr virus latent membrane protein 1. Magnification was 200.

a

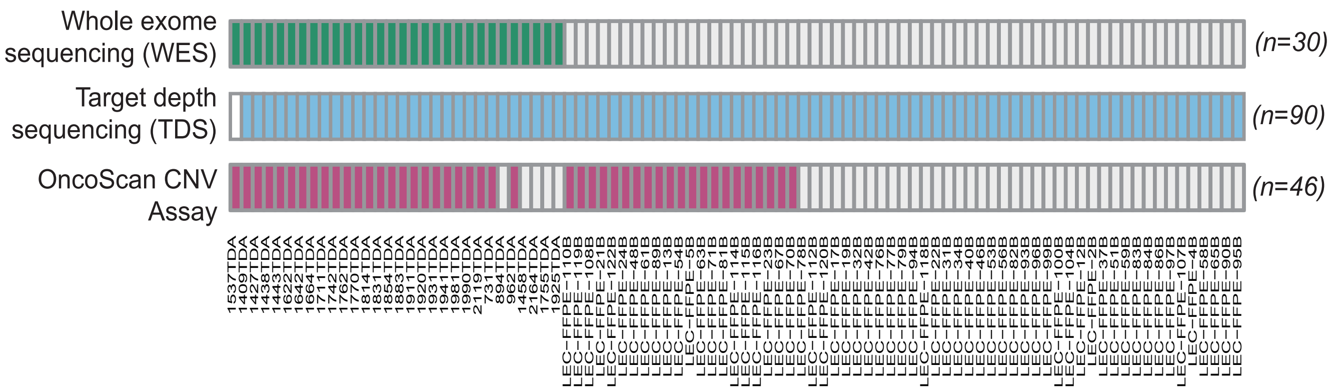

b

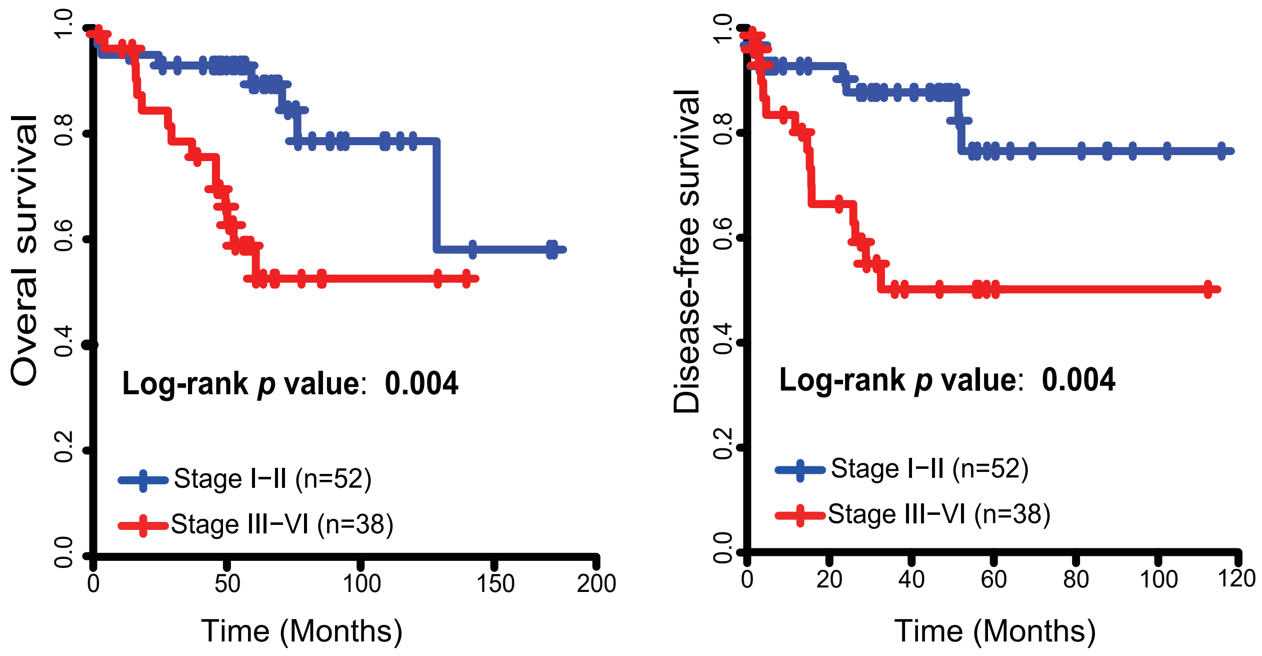

Supplementary Figure 2. Clinical characterization and sample profiling for 91 patients with pulmonary LELC. a, Columns represent samples while rows correspond to platforms. **b,** Kaplan-Meier survival curves for patients with pulmonary LELC by TNM stage (excluded 1 patients with mising TNM stage information). LELC, lymphoepithelioma-like carcinoma; TNM, tumor, lymph node, metastasis; CNV, copy number variation.

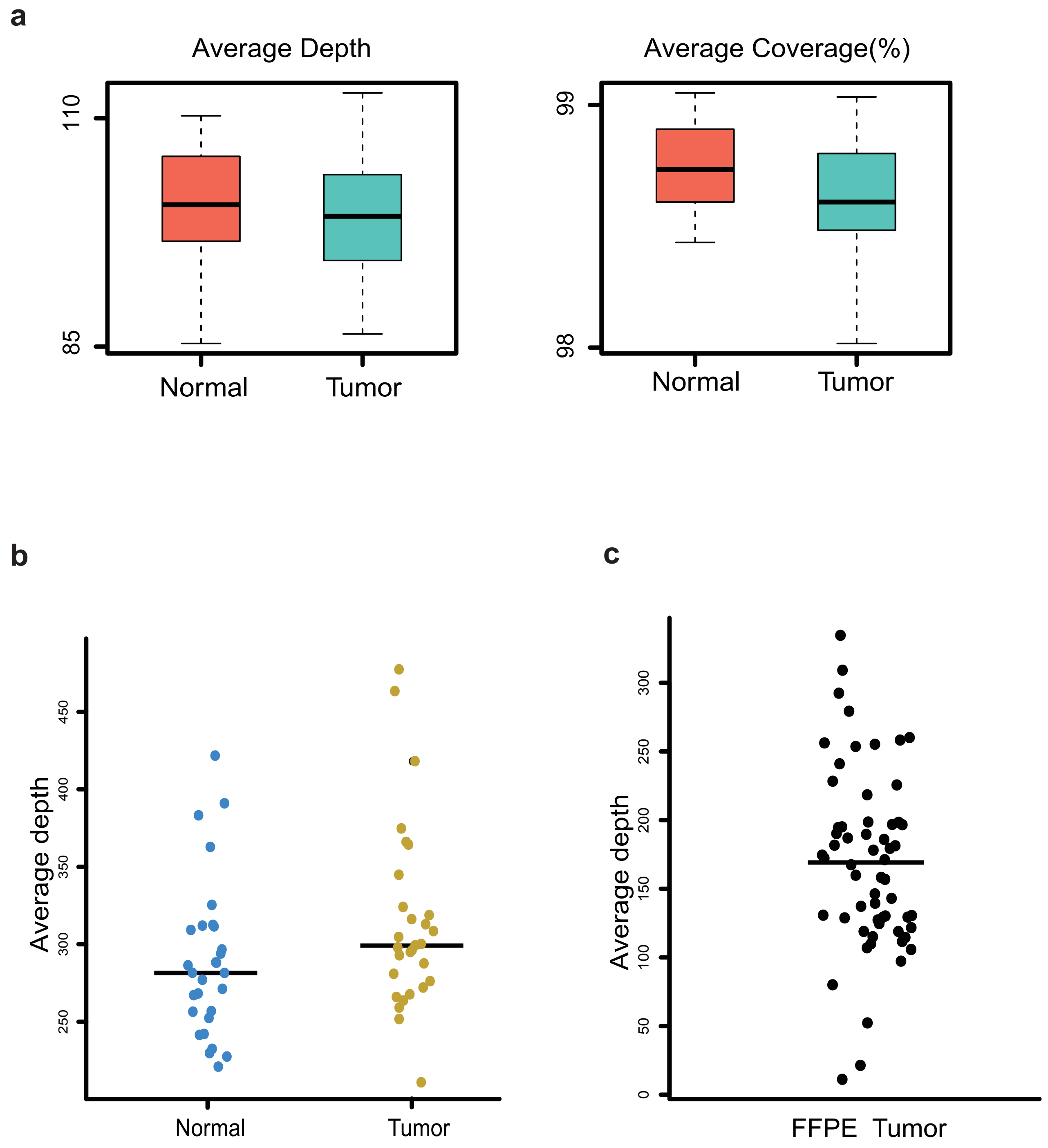

Supplementary Figure 3. Sequencing depth and coverage of 91 pulmonary LELC patients. a, Box plot of mean depth and coverage of 30 tumor and matched adjacent normal pairs for WES. **b,** Mean depth of 29 tumor and matched adjacent normal pairs for TDS. **c,** Mean depth of 61 FFPE tumors for TDS. Black lines in the boxplot and scatter plot of **a, b, and c** denote the median depth and coverage, respectively. Upper and lower lines in **a** denote ranges. LELC, lymphoepithelioma-like carcinoma; WES, whole exon sequencing; TDS, targeted deep sequencing; FFPE, formalin-fixed, paraffin-embedded.

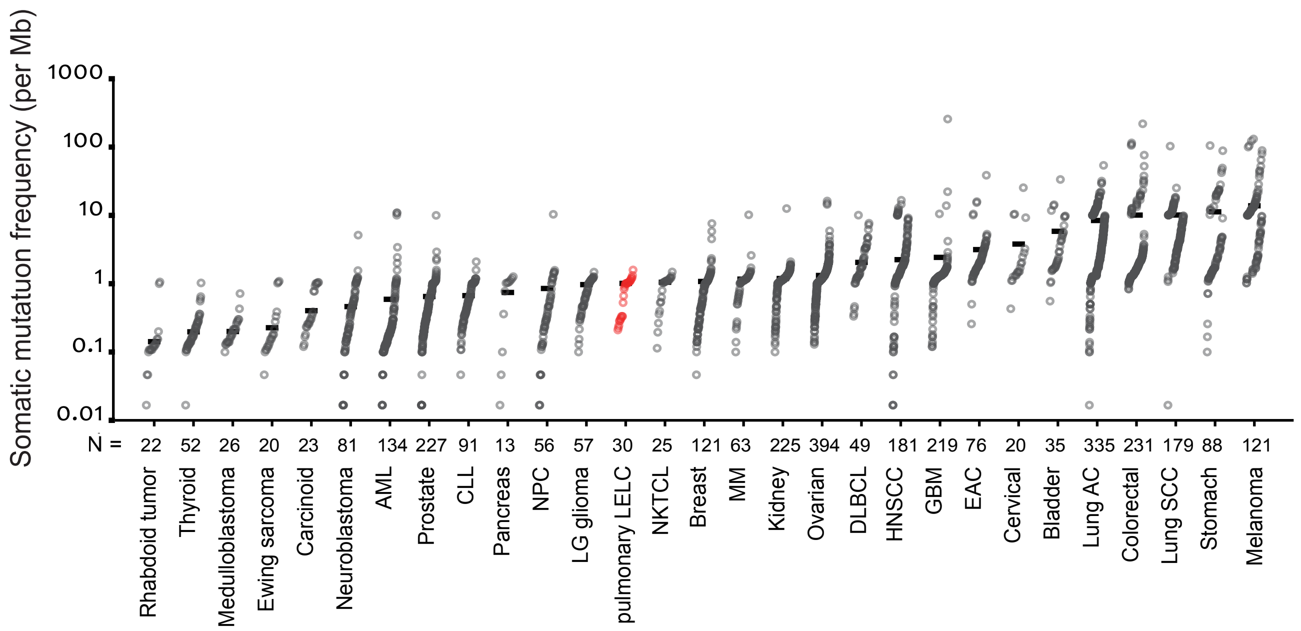

Supplementary Figure 4. Somatic mutation rates detected in exomes from pulmonary LELC and other human cancers. Each dot represents an examined tumor sample, and tumor types are ordered by their mean somatic mutation rates (indicated by the black dash line). The number beneath X-axis indicates the number of examined cases of each cancer while Y-axis is the frequency of somatic mutations per Mb. Except pulmonary LELC and NKTCL, all of the other cancers are adopted from Lin, D. C. et al (see refs: 1). AML, acute myeloid leukemia; CLL, chronic lymphocytic leukemia; NPC, nasopharyngeal carcinoma;LELC, lymphoepithelioma-like carcinoma; NKTCL, natural killer/T cell lymphoma; MM, multiple myeloma; DLBCL, diffuse large B-cell lymphoma; HNSCC, head and neck squamous cell carcinoma; GBM, glioblastoma multiforme; EAC, esophageal adenocarcinoma; Lung AC, lung adenocarcinoma; Lung SCC, lung squamous cell carcinoma correspond to platforms; Mb, megabase.

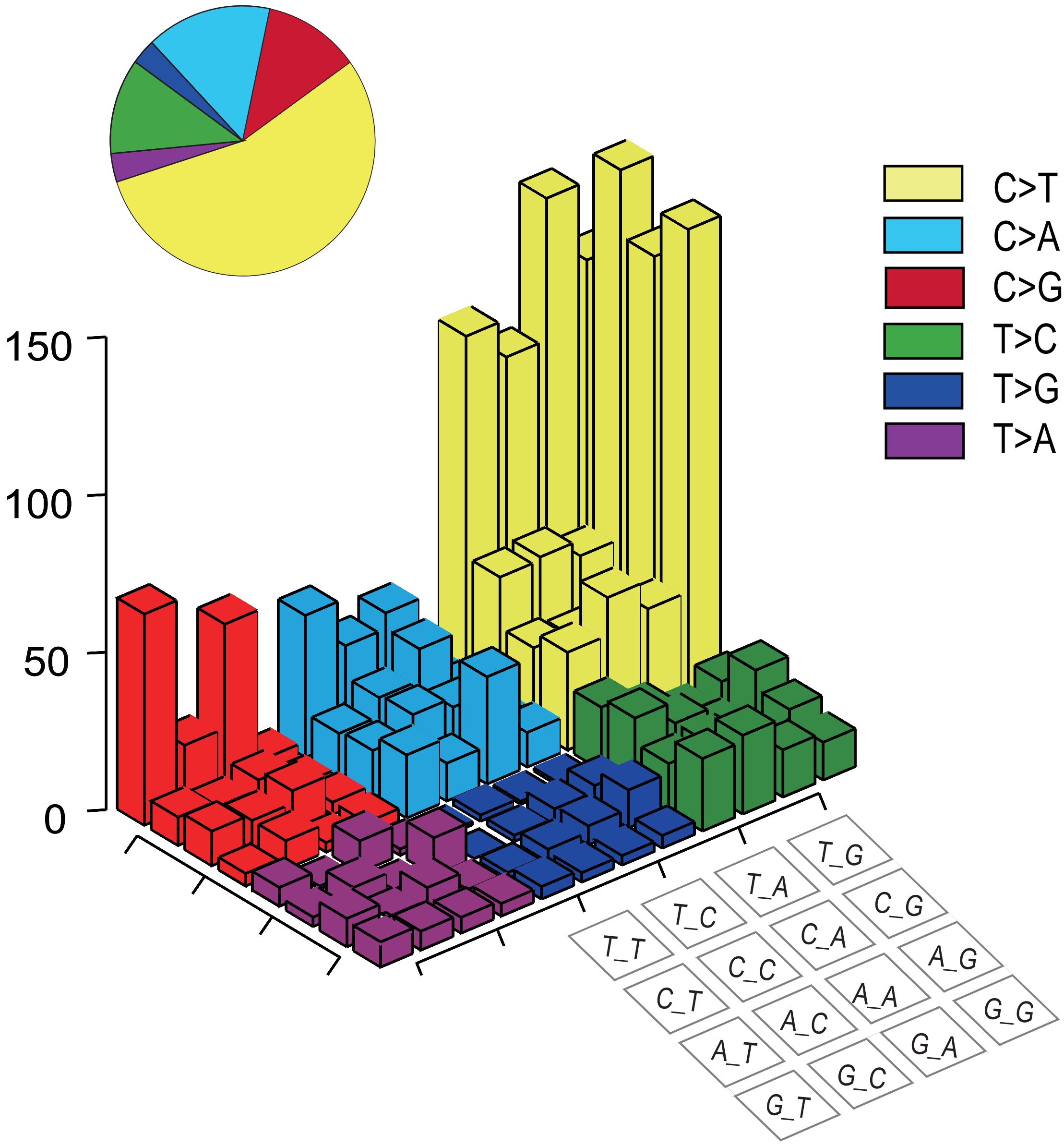

Supplementary Figure 5. Mutation spectrum analysis of pulmonary LELC. Mutation spectrum is calculated from 30 pulmonary LELC samples subjected to WES. Base substitutions are divided into 96 subtypes based on mutation type and nucleotides flanking the mutated base. The height of the bar represents the somatic mutation number of each substitution. LELC, lymphoepithelioma-like carcinoma.

a

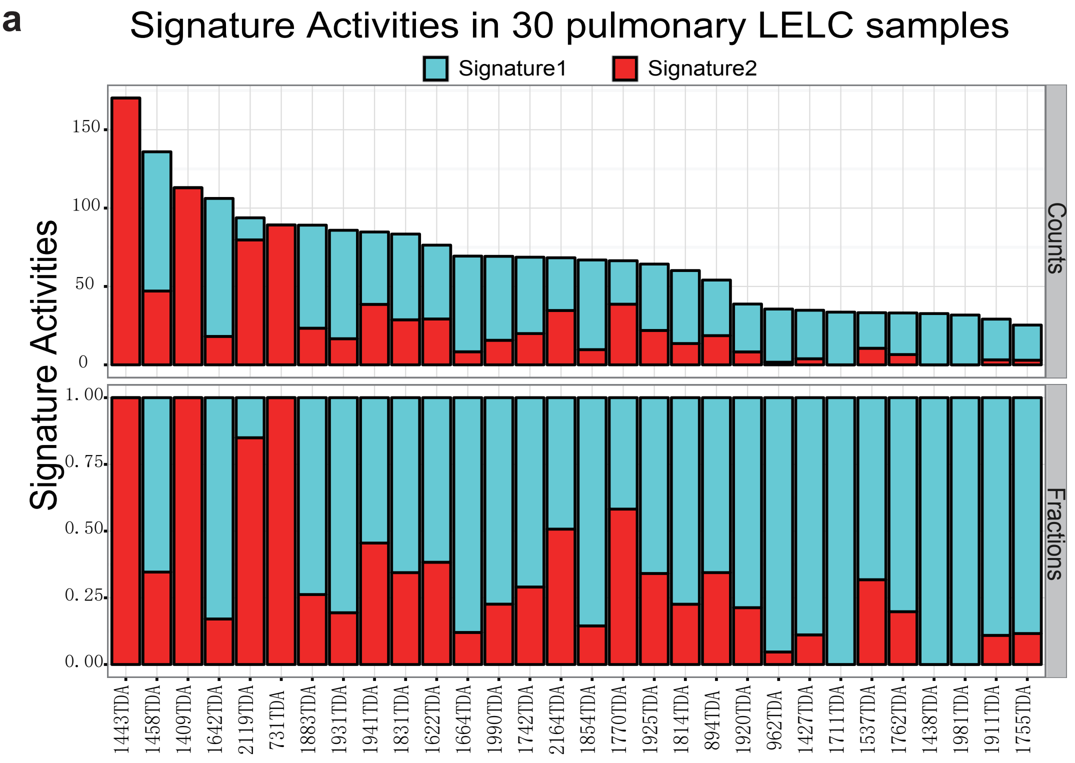

Signature Activities in 30 pulmonary LELC samples
Signature1
Signature2
Signature Activities
Counts
Fractions
150
100
50
0
1.00
0.75
0.50
0.25
0.00
1443TDA
1458TDA
1409TDA
1642TDA
2119TDA
731TDA
1883TDA
1931TDA
1941TDA
1831TDA
1622TDA
1664TDA
1990TDA
1742TDA
2164TDA
1854TDA
1770TDA
1925TDA
1814TDA
894TDA
1920TDA
962TDA
1427TDA
1711TDA
1537TDA
1762TDA
1438TDA
1981TDA
1911TDA
1755TDA

b

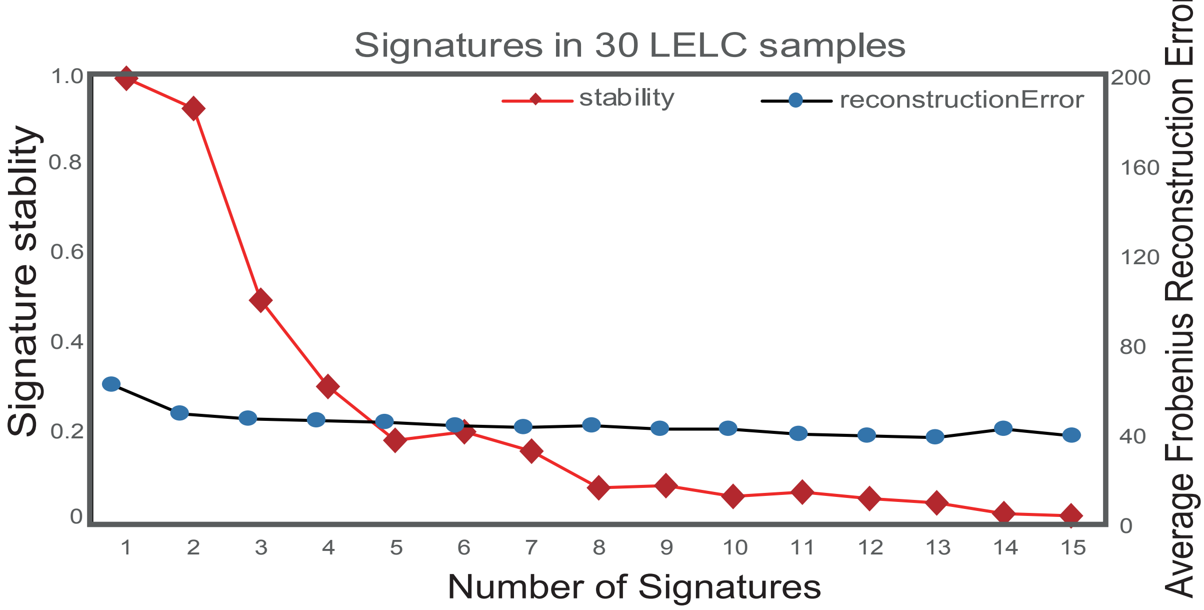

Signatures in 30 LELC samples
stability
reconstructionError
Signature stablity
Average Frobenius Reconstruction Error
Number of Signatures
1.0
0.8
0.6
0.4
0.2
0
200
160
120
80
40
0
1 2 3 4 5 6 7 8 9 10 11 12 13 14 15

c

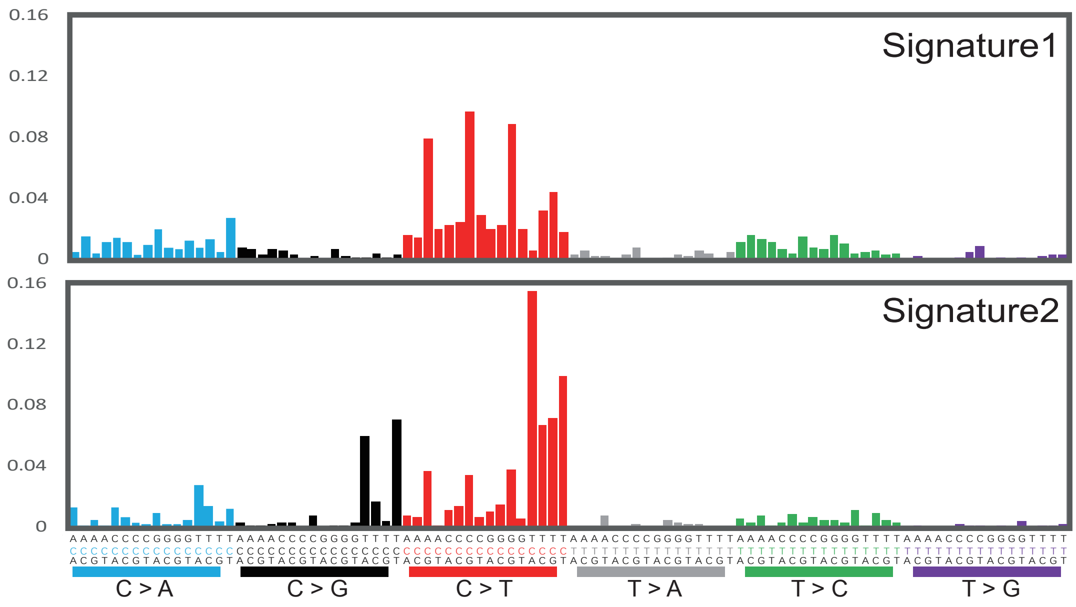

Signature1
Signature2
0.16
0.12
0.08
0.04
0
C > A
C > G
C > T
T > A
T > C
T > G

d

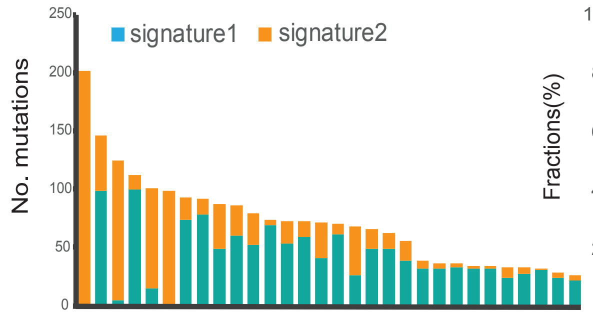

signature1
signature2
No. mutations
250
200
150
100
50
0

e

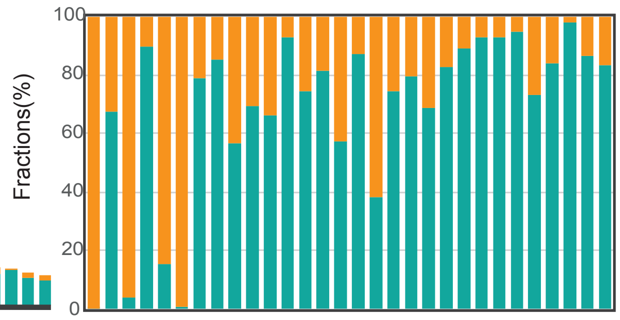

Fractions(%)
100
80
60
40
20
0

f

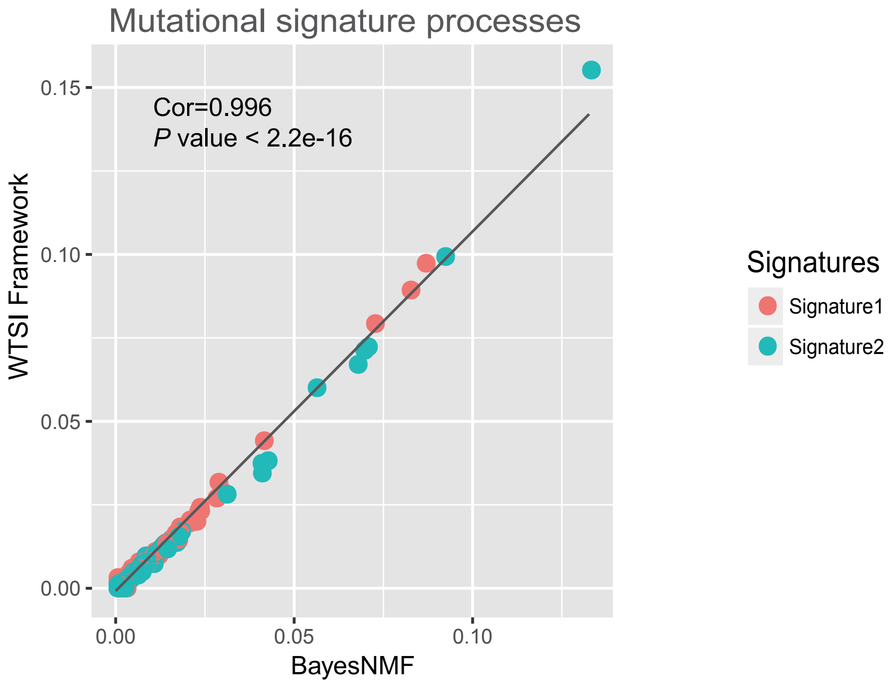

Mutational signature processes
Cor=0.996
P value < 2.2e-16
WTSI Framework
BayesNMF
0.00
0.05
0.10
0.15
Signatures
Signature1
Signature2

g

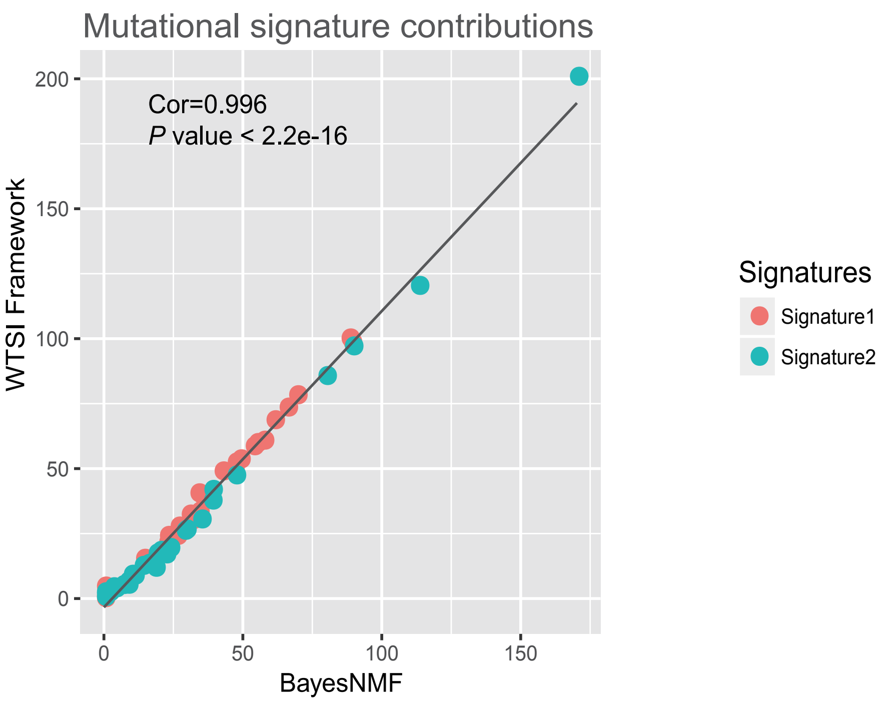

Supplementary Figure 6. Mutational signature analysis. a, Contributions of mutational signatures to each pulmonary LELC patients identified by the BayesNMF algorithm (BayesNMF). **b,** The signature stability and reconstruction error are estimated by the NMF methodology described by Alexandrov *et al* (WTSI framework). Two signatures give the optimal trade-off between signature stability and reconstruction error and are thus selected. **c,** Each signature is displayed according to the 96 substitution classification defined by the substitution class and sequence context. The X-axis shows mutation types and the Y-axis shows trinucleotide frequency of each mutation type. **d, e,** Contributions of mutational signatures to each pulmonary LELC patient (WTSI framework). **f, g,** Scatter plot showing the correlation of mutational processes and contributions between BayesNMF algorithm (BayesNMF) and WTSI framework . The p value is calculated by Pearson correlation. LELC, lymphoepithelioma-like carcinoma.

a

Pulmonary LELC gene N=76

TP53	TRAF3	DDX11	CGREF1	MADCAM1	MUC4
SPDEF	CLTC	USP29	ABL2	BCL10	CRTC3
CD209	HIVEP1	EPHB1	FMN2	HECTD1	HRAS
DPF3	MGA	PTPRG	PTPRO	SALL3	SUPT16H
FOLH1	JAK2	MYO9B	NCOR1	RGPD5	ZNF521
FGFR3	LAMB1	MAP3K1	MET	KTM2B	RB1
LAMA4	POLE	MYOCD	NOTCH1	NSD1	PRDM16
MSN	ALPP	PPM1D	SMC3	SPEN	XPO1
CARD14	DRAM2	EP300	KDM 6A	CSF3R	BTRC
RASA1	PTPRD	FBXO11	LAMA2	CYLD	C12orf51
SAMD9	RGS3	HOXA11	RGPD8	TMPRSS13	RNF212
RHPN2	CUL4B	IQGAP2	CBLB	BIRC6	USP6
PRKD2	ZFHX3	VHL	DDX10		

NF-κB gene N=14

TRAF1	TRAF2
CARD11	MALT1
CARD10	MAP3K14
RELA	RELB
TRAF6	TRADD
NFKB1	NFKBIA
CHUK	IKBKB

LUAD gene N=10

TP53	KRAS
STK11	KEAP1
NF1	BRAF
RB1	EGFR
PIK3CA	ATM

NPC gene N=12

TP53	TSHZ3
PIK3CA	ERBB3
KRAS	KMT2C
NRAS	KMT2D
BAP1	FGFR2
ERBB2	ARID1A

LUSC gene N=10

TP53	KEAP1
NOTCH1	MLL2
PTEN	EGFR
KRAS	RB1
NF1	PIK3CA

NKT gene N=10

TP53	MGA
STAT3	STAT5B
DDX3X	EP300
NRAS	KMT2D
MSN	ASXL3

SCLC gene N=4

TP53	RGS7
RB1	FPR1

Total Unique Gene Number = 114

b

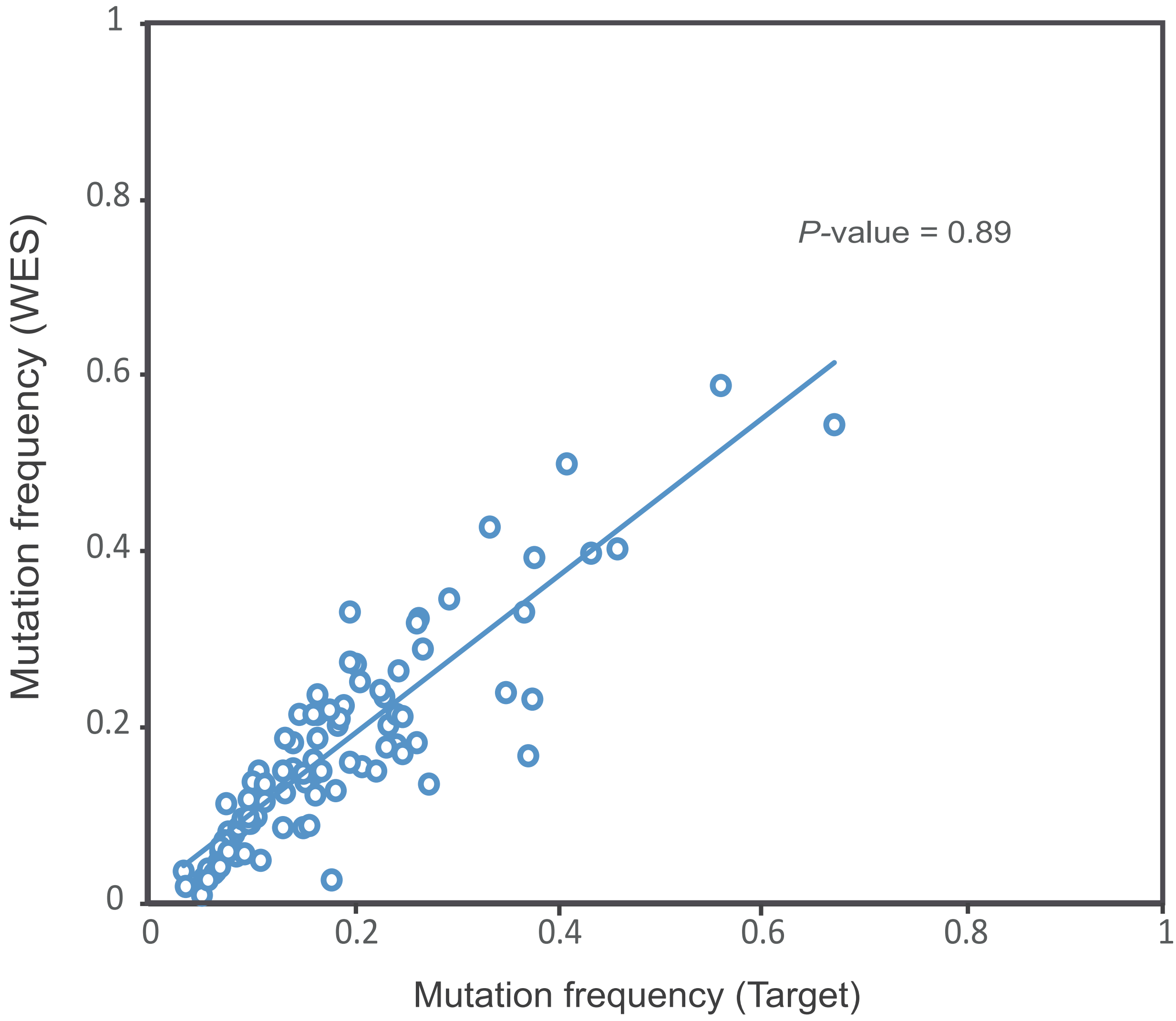

Supplementary Figure 7. Validated genes and mutation frequency validation for SNVs by TDS. a, Genes select for pulmonary LELC and other cancer types . **b,** Pearson correlation of mutation allele frequency between TDS and WES. Each dot represents a SNV. X-axis and Y-axis denote the mutation frequency of each SNVs in TDS data and WES. Mutation frequency is calculated by (variant supporting reads)/(total depth) in the SNV site. LELC, lymphoepithelioma-like carcinoma; NPC, nasopharyngeal carcinoma; NKTCL, natural killer/T cell lymphoma; LUAD, lung adenocarcinoma; SCLC, small-cell lung carcinoma; SNVs, single nucleotide variants; TDS, targeted deep sequencing.

a

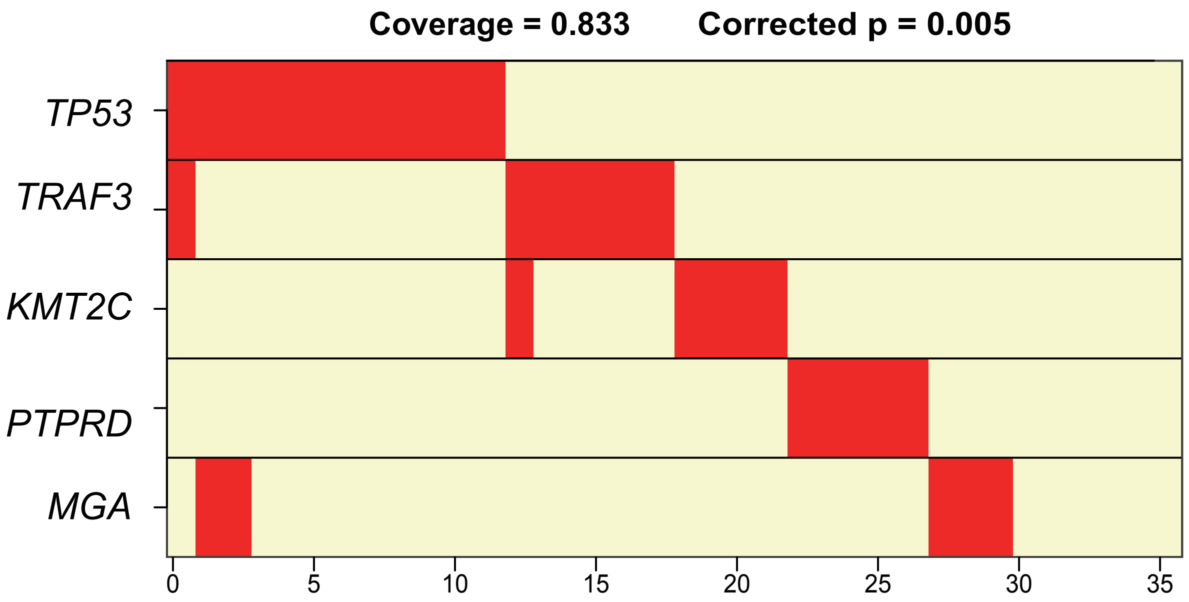

Coverage = 0.833
Corrected p = 0.005
TP53
TRAF3
KMT2C
PTPRD
MGA
0
5
10
15
20
25
30
35

b

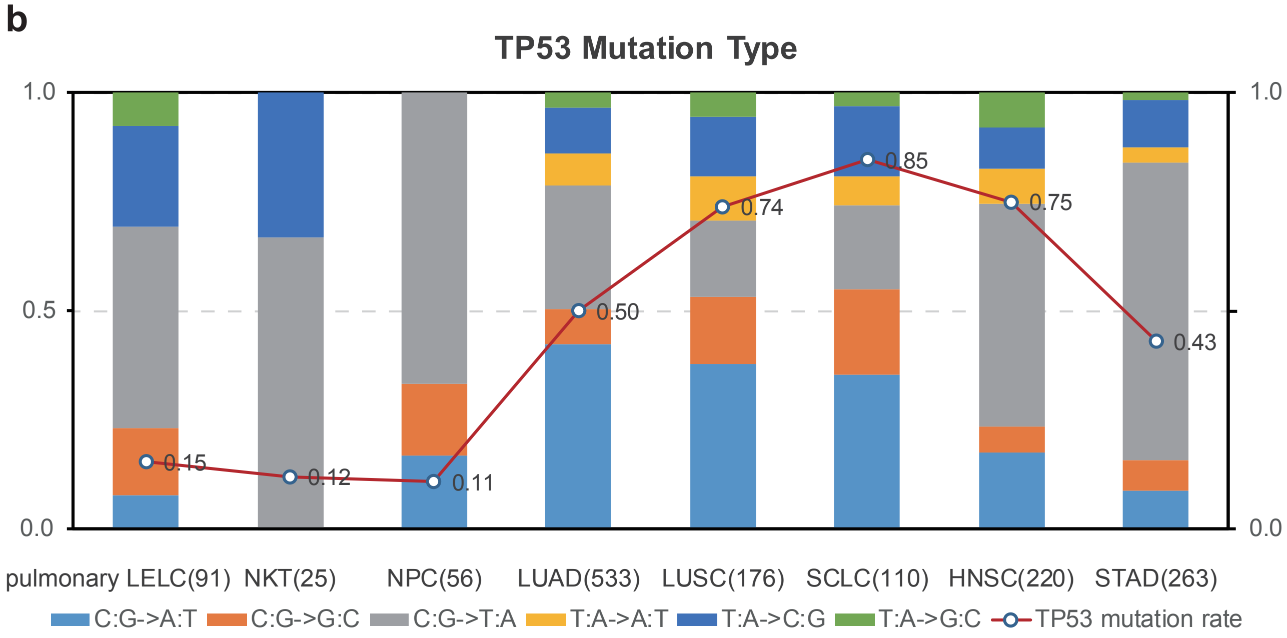

TP53 Mutation Type
1.0
0.5
0.0
0.15
0.12
0.11
0.50
0.74
0.85
0.75
0.43
pulmonary LELC(91)
NKT(25)
NPC(56)
LUAD(533)
LUSC(176)
SCLC(110)
HNSC(220)
STAD(263)
C:G->A:T
C:G->G:C
C:G->T:A
T:A->A:T
T:A->C:G
T:A->G:C
TP53 mutation rate

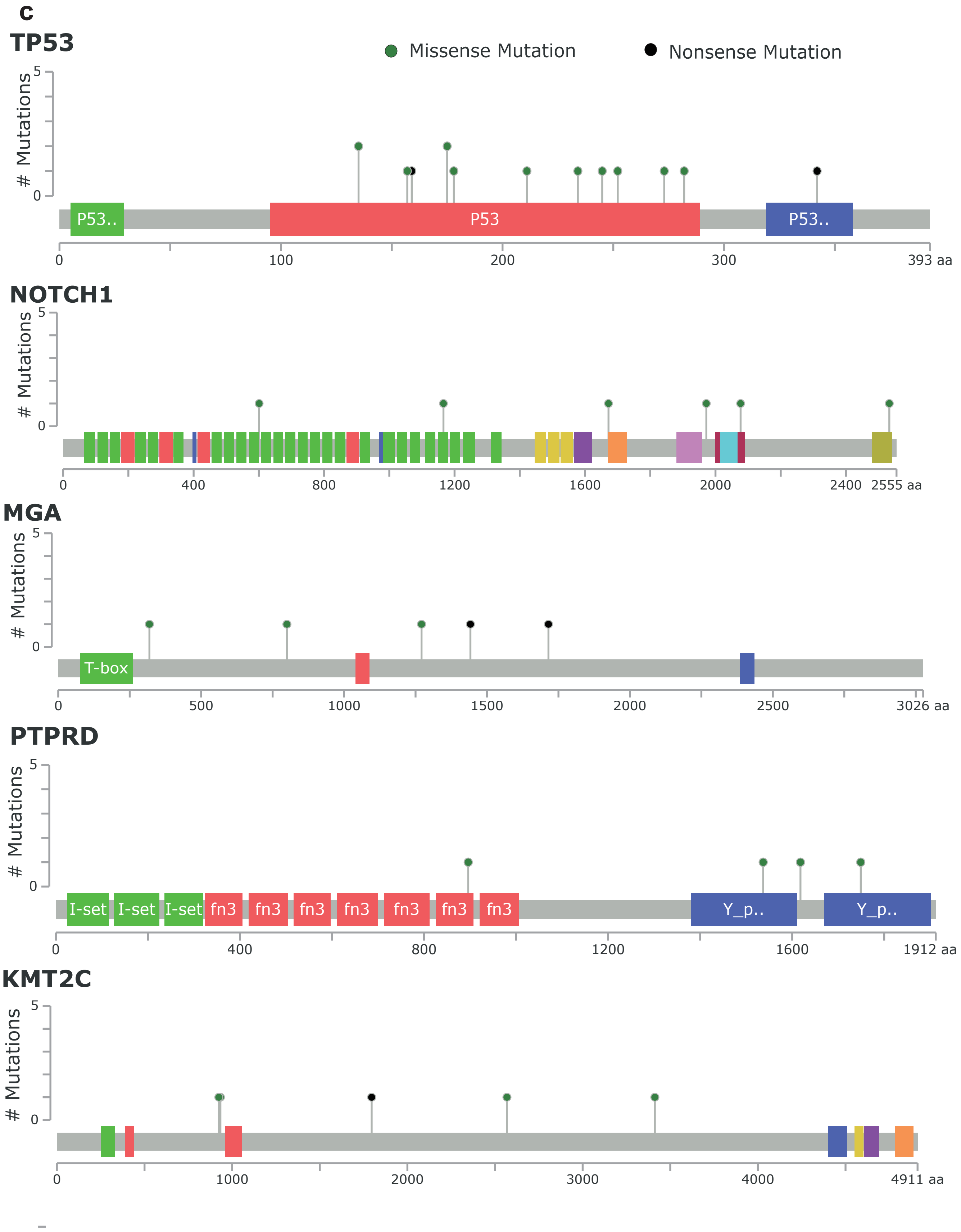
c
TP53
Missense Mutation
Nonsense Mutation
Mutations
P53..
P53
P53..
0
100
200
300
393 aa
NOTCH1
2400
2555 aa
MGA
T-box
3026 aa
PTPRD
I-set
fn3
Y_p..
1912 aa
KMT2C
4911 aa

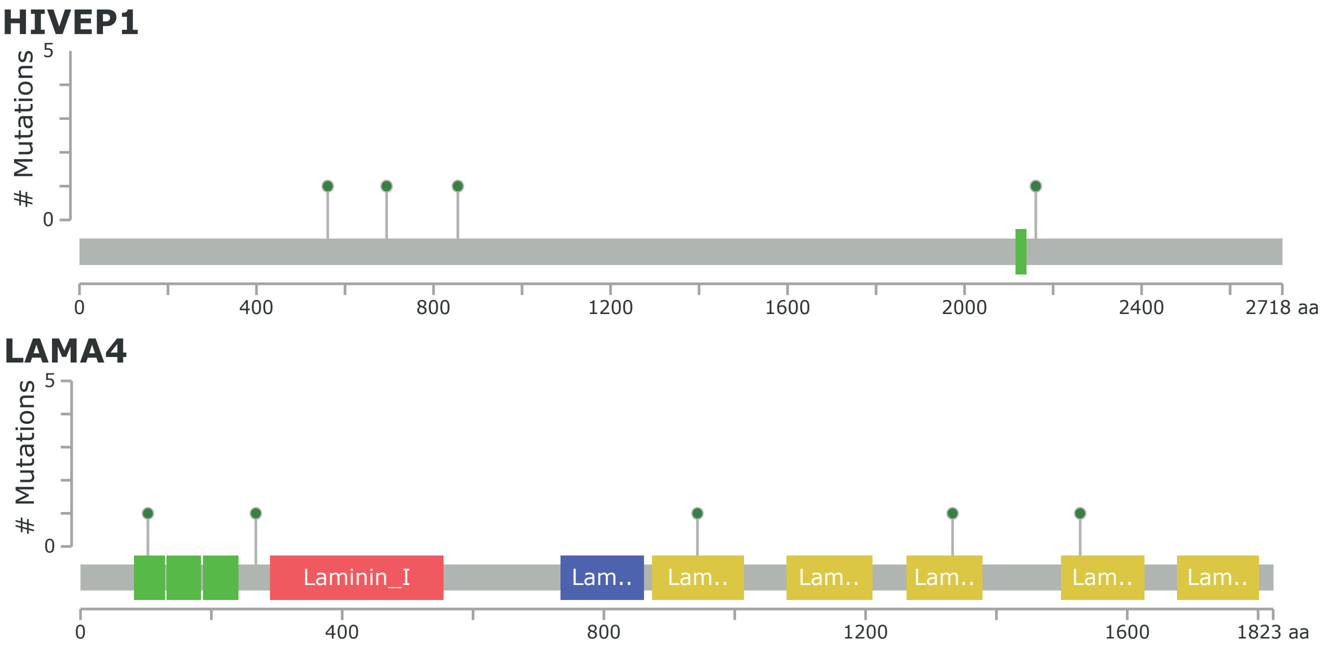

d

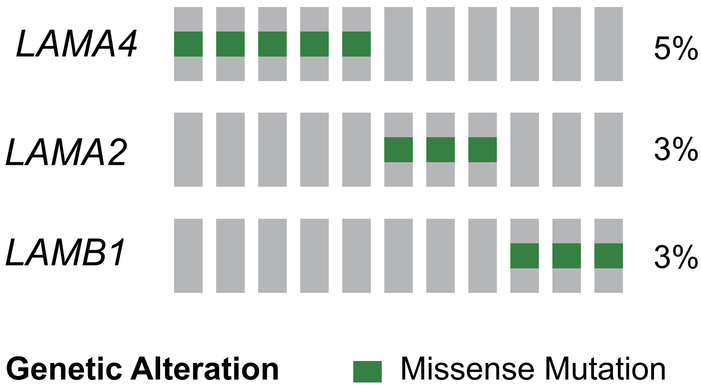

Supplementary Figure 8. Characterization of recurrent mutations in pulmonary LELC. a, Mutually exclusive mutations are observed in the frequently mutated genes by using MEGSA (see refs: 2 ; download from https://dceg.cancer.gov/tools/analysis/megsa). For each mutated gene (row) indicated, tumors (columns) with or without mutations are labeled in red or gray, respectively. **b,** Mutation spectrum (six mutation types) of TP53 mutations in eight cancer types. Except pulmonary LELC, NPC, NKT and SCLC, all mutation data of the other cancers are downloaded from The Cancer Genome Atlas (TCGA GDAC) database (http://gdac.broadinstitute.org/runs/analyses__2016_01_28/reports/cancer/). **c,** Mutation types and relative positions of somatic mutations are shown (generated by MutationMapper, http://www.cbioportal.org/mutation_mapper.jsp) in the transcript of each mutated gene. **d ,** An oncoprint view of laminins family gene mutation status in pulmonary LELC. NPC, nasopharyngeal carcinoma; LELC, lymphoepithelioma-like carcinoma; NKT, natural killer/T cell lymphoma; HNSC, head and neck squamous cell carcinoma; LUAD, lung adenocarcinoma; LUSC, lung squamous cell carcinoma; SCLC, small-cell lung carcinoma; STAD, stomach adenocarcinoma.

a

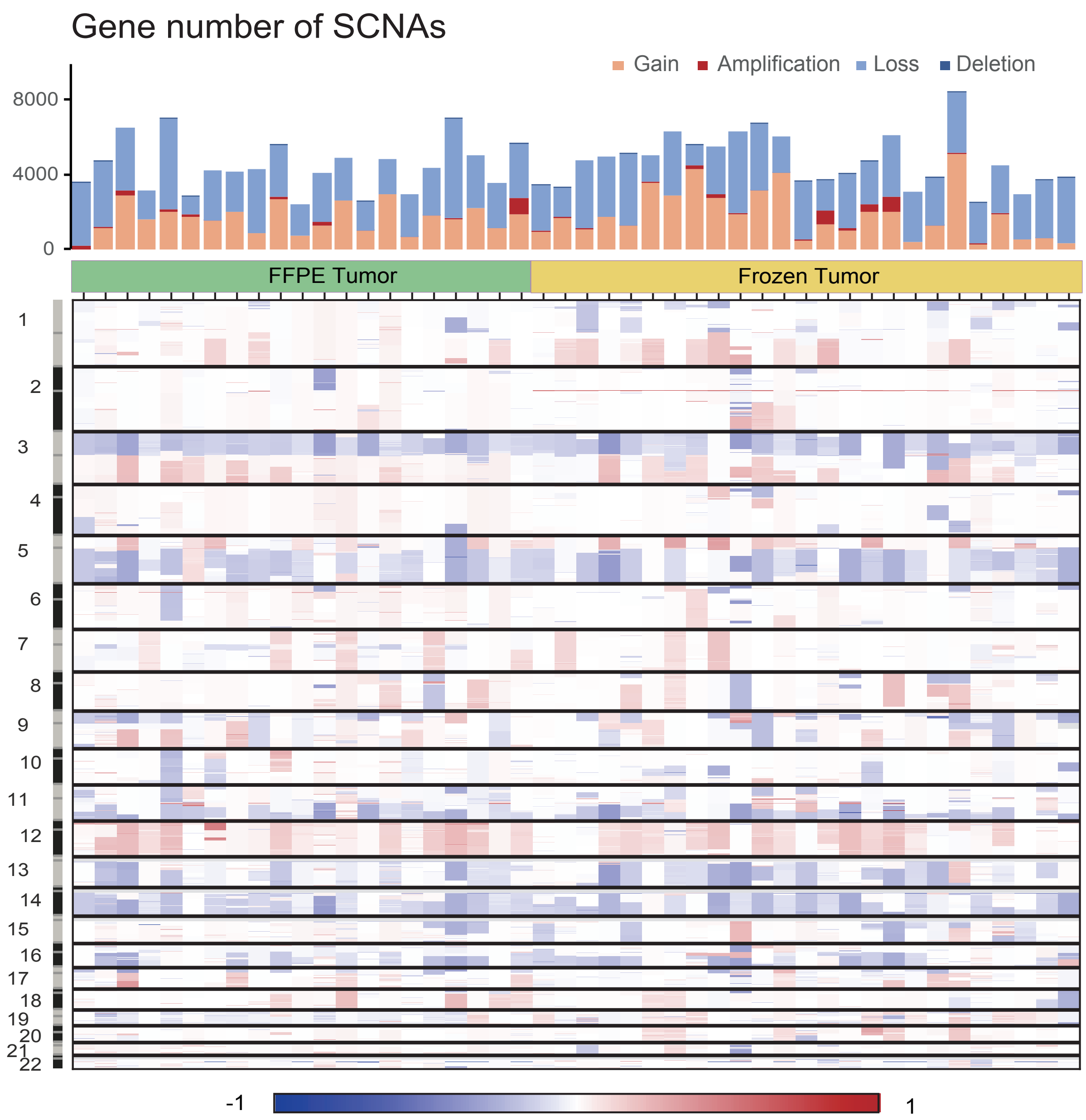

Gene number of SCNAs
Gain
Amplification
Loss
Deletion
8000
4000
0
FFPE Tumor
Frozen Tumor
1
2
3
4
5
6
7
8
9
10
11
12
13
14
15
16
17
18
19
20
21
22
-1
1

b

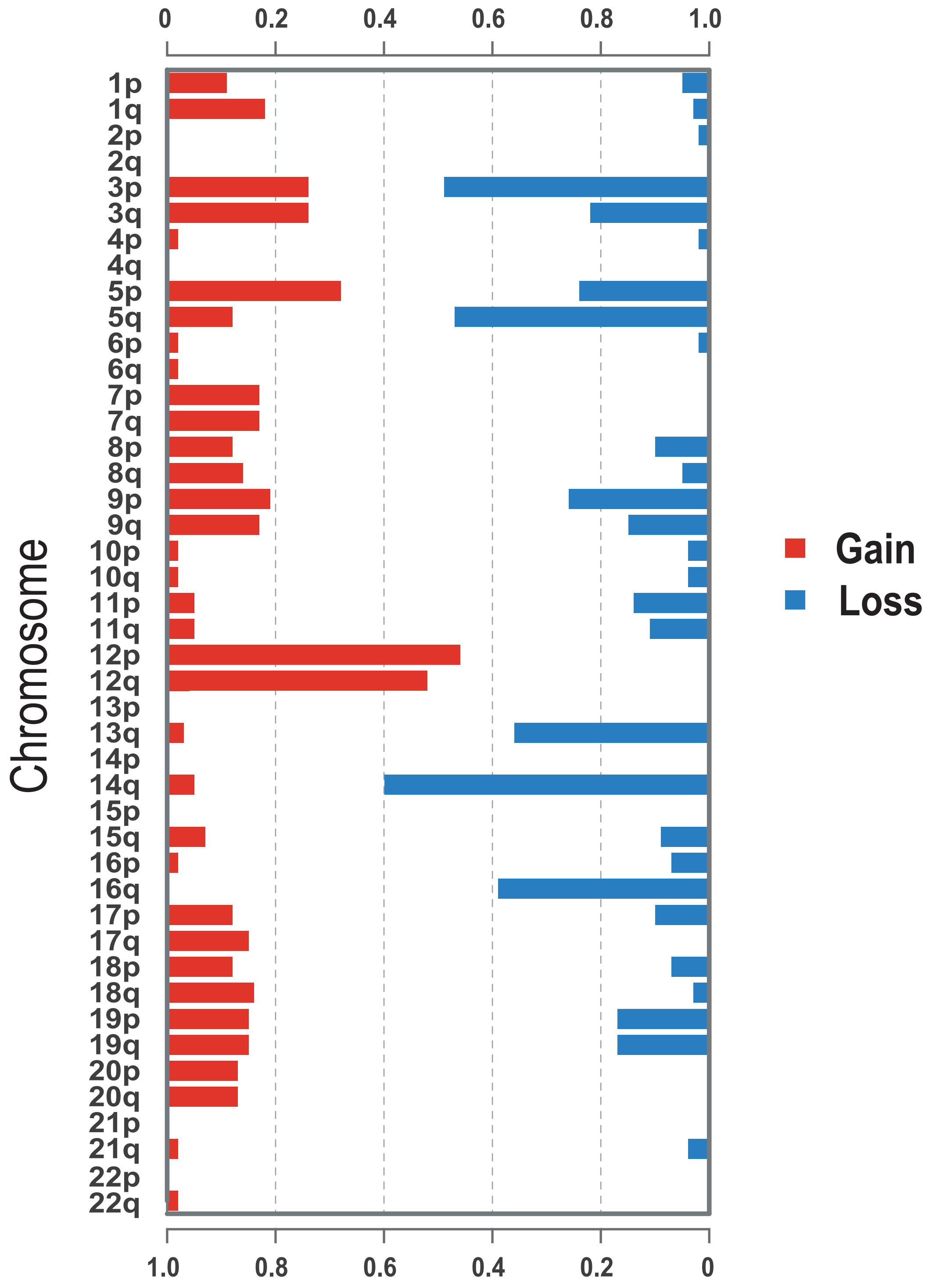

Supplementary Figure 9. Somatic copy number profiling of 46 patients with pulmonary LELC. a, Copy number alterations for 25 frozen tumors and 21 FFPE tumors are displayed on global chromosomal gains (shown in red) and losses (shown in blue). **b,** Frequency of broad somatic copy number alterations (SCNAs) across 46 pulmonary LELC tumors. Chromosomal arm regions with copy gain (red) and loss (blue) are shown. LELC, lymphoepithelioma-like carcinoma; FFPE, formalin-fixed paraffin-embedded.

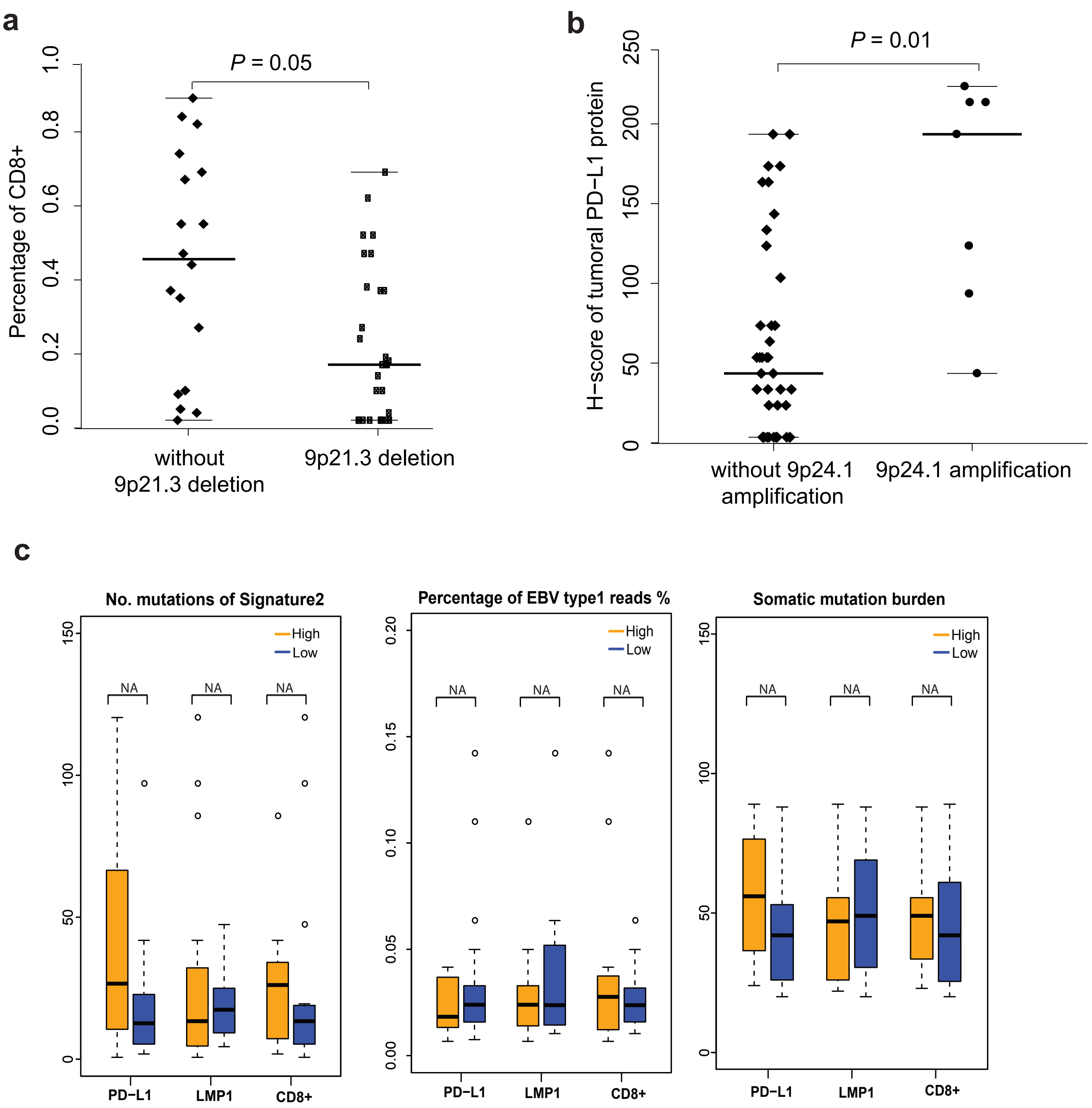

Supplementary Figure 10. Immune Micromanagement and Key Genomic Alterations. **a**, Association between 9p21.3 deletion and percentage of CD8 positive tumor infiltrating lymphocytes (TILs). **b**, Association between 9p24.1 amplification and tumoral PD-L1 expression level. Bold black lines in the scatter plot of **a, b** denote the median value. Upper and lower black lines denote the range of values. **c**, Boxplot to display the association between PDL1, LMP1, CD8+ TILs and key genomic alterations (signature 2; EBV type 1 reads; somatic mutation burden). Black lines in the boxplot denote the median value. Upper and lower lines denote the ranges of PDL1, LMP1, CD8+ TILs with outliers are shown as dots; All the statistical tests are Student's t-tests.

a

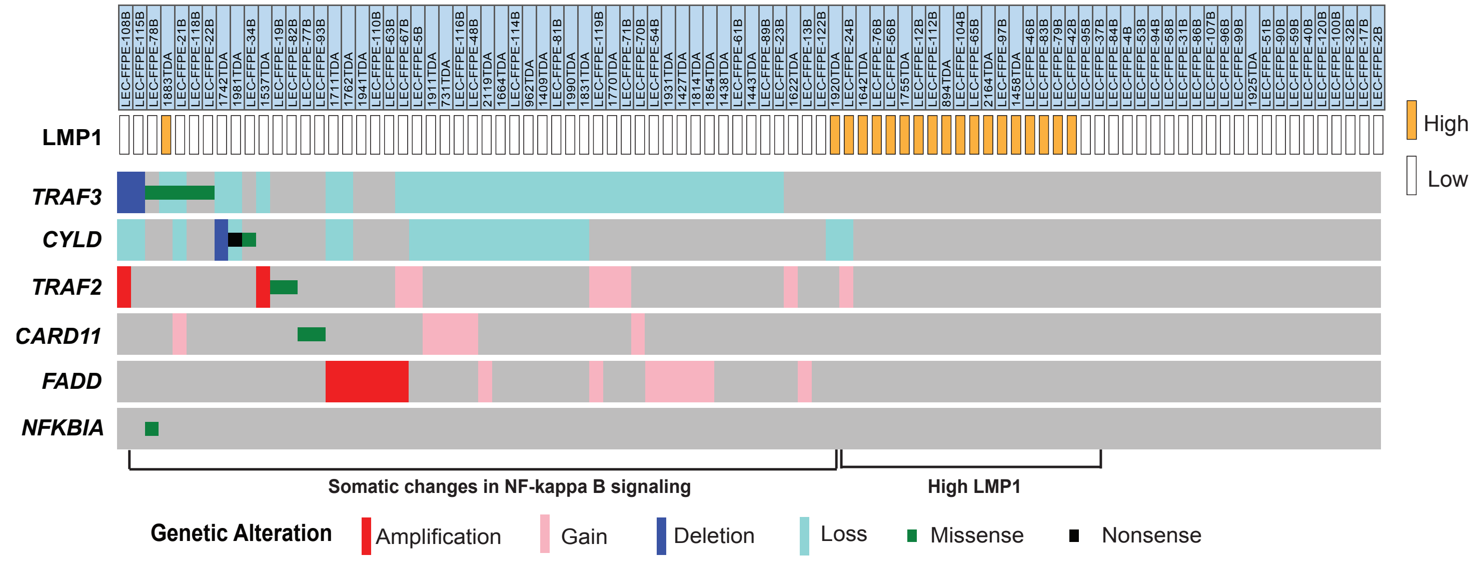

b

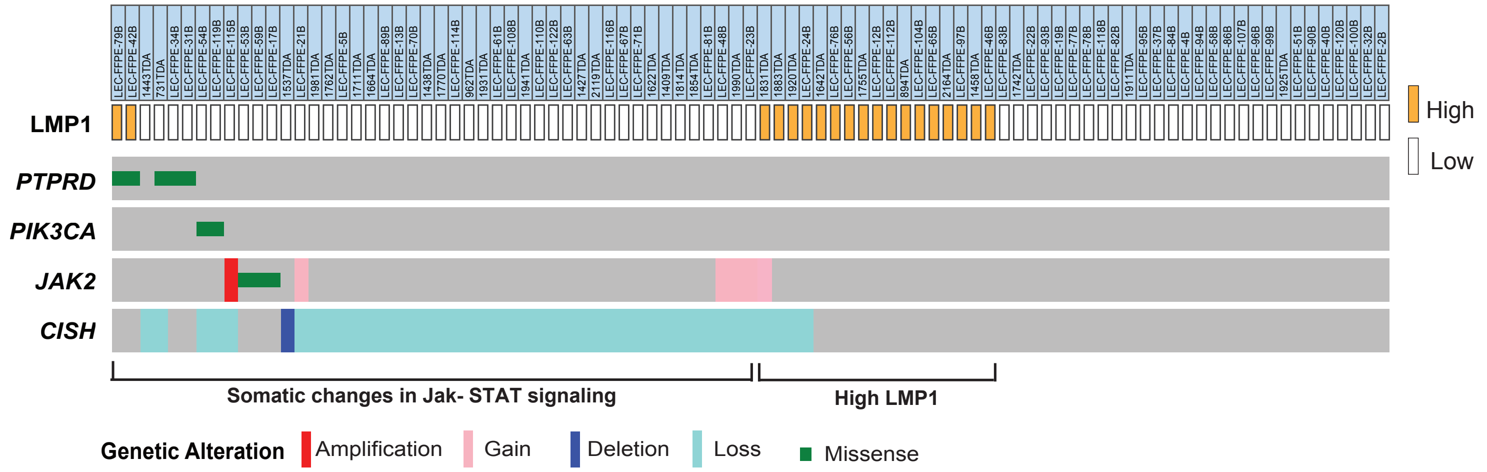

c

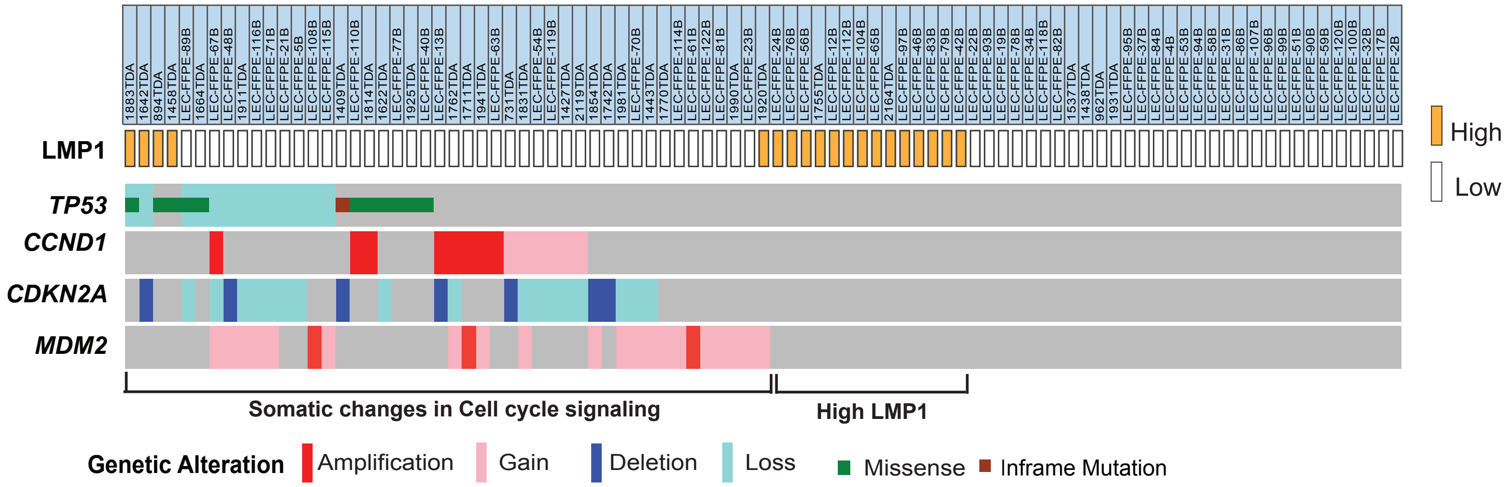

Supplementary Figure 11. Genomic aberrations in three core pathways in pulmonary LELC and LMP1 overexpression. a, NF-κB pathway; **b**, JAK/STAT pathway; **c**, cell cycle pathway. LMP1 expression was categorized by Hscore of 100.

a

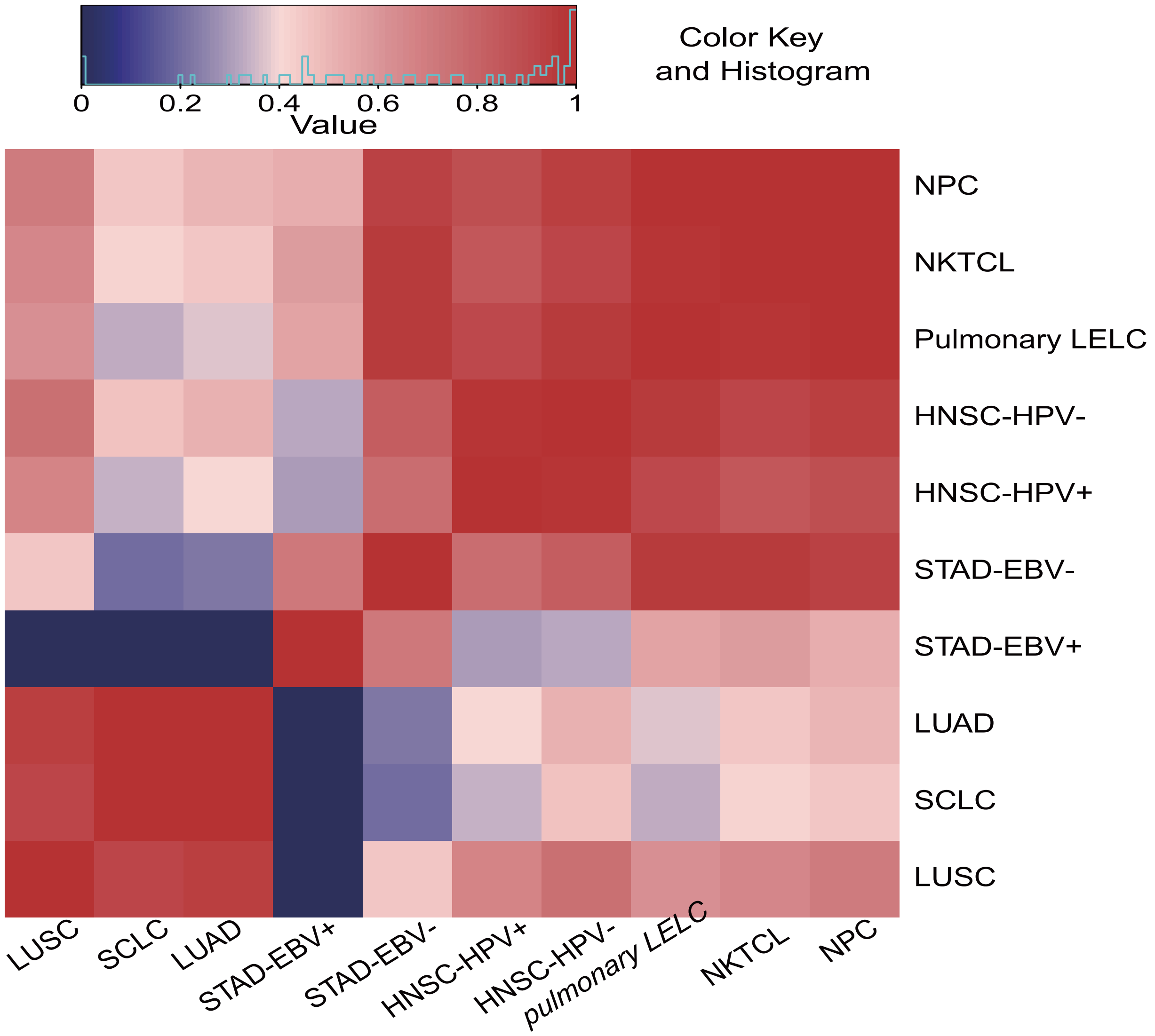
Color Key
and Histogram
0
0.2
0.4
0.6
0.8
1
Value
NPC
NKTCL
Pulmonary LELC
HNSC-HPV-
HNSC-HPV+
STAD-EBV-
STAD-EBV+
LUAD
SCLC
LUSC
LUSC
SCLC
LUAD
STAD-EBV+
STAD-EBV-
HNSC-HPV+
HNSC-HPV-
pulmonary LELC
NKTCL
NPC

b

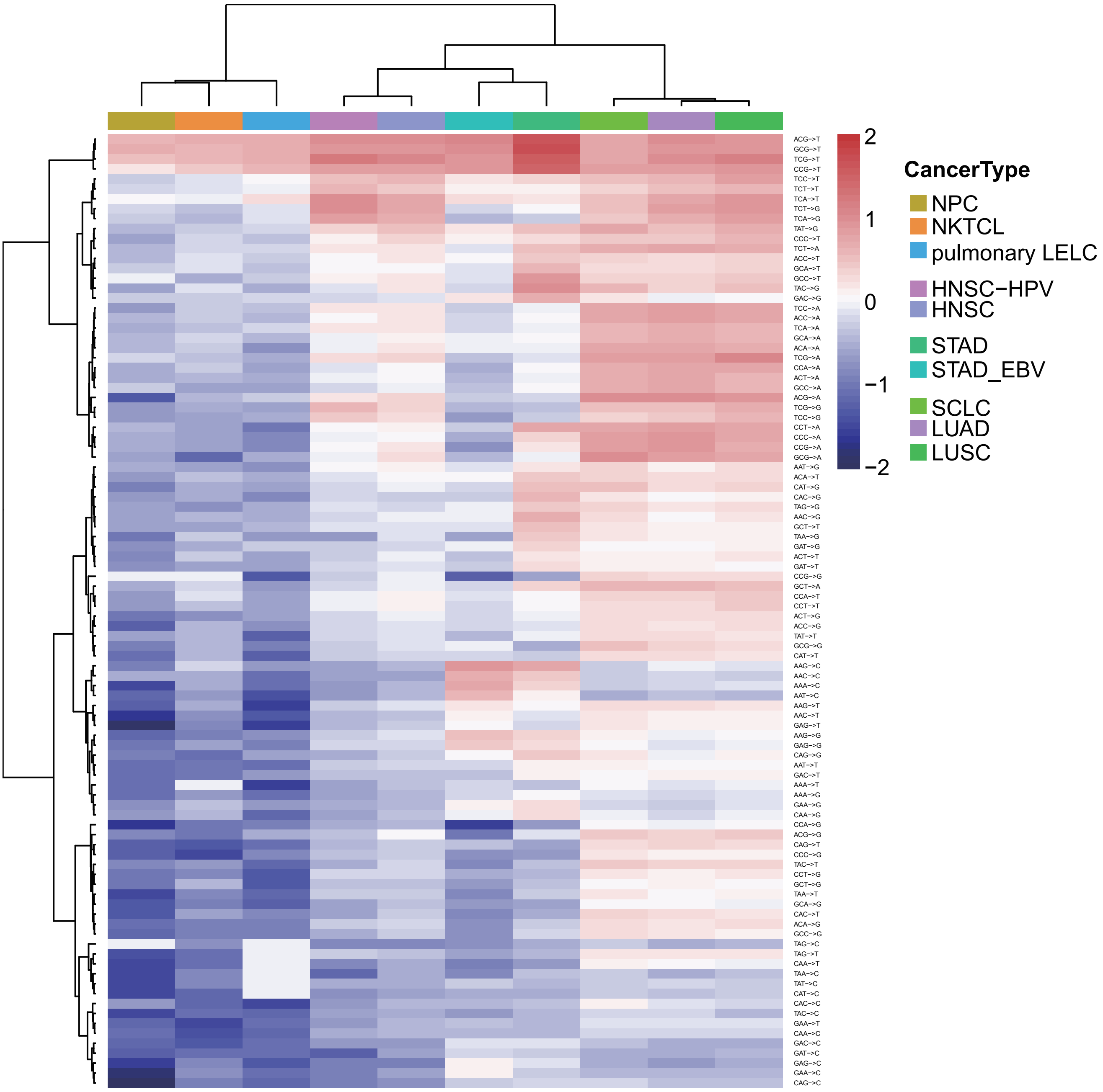
ACG->T
GCG->T
TCG->T
CCG->T
TCC->T
TCT->T
TCA->T
TCT->G
TCA->G
TAT->G
CCC->T
TCT->A
ACC->T
GCA->T
GCC->T
TAC->G
GAC->G
TCC->A
ACC->A
TCA->A
GCA->A
ACA->A
TCG->A
CCA->A
ACT->A
GCC->A
ACG->A
TCG->G
TCC->G
CCT->A
CCC->A
CCG->A
GCG->A
AAT->G
ACA->T
CAT->G
CAC->G
TAG->G
AAC->G
GCT->T
TAA->G
GAT->G
ACT->T
GAT->T
CCG->G
GCT->A
CCA->T
CCT->T
ACT->G
ACC->G
TAT->T
GCG->G
CAT->T
AAG->C
AAC->C
AAA->C
AAT->C
AAG->T
AAC->T
GAG->T
AAG->G
GAG->G
CAG->G
AAT->T
GAC->T
AAA->T
AAA->G
GAA->G
CAA->G
CCA->G
ACG->G
CAG->T
CCC->G
TAC->T
CCT->G
GCT->G
TAA->T
GCA->G
CAC->T
ACA->G
GCC->G
TAG->C
TAG->T
CAA->T
TAA->C
TAT->C
CAT->C
CAC->C
TAC->C
GAA->T
CAA->C
GAC->C
GAT->C
GAG->C
GAA->C
CAG->C
2
1
0
−1
−2
CancerType
NPC
NKTCL
pulmonary LELC
HNSC−HPV
HNSC
STAD
STAD_EBV
SCLC
LUAD
LUSC

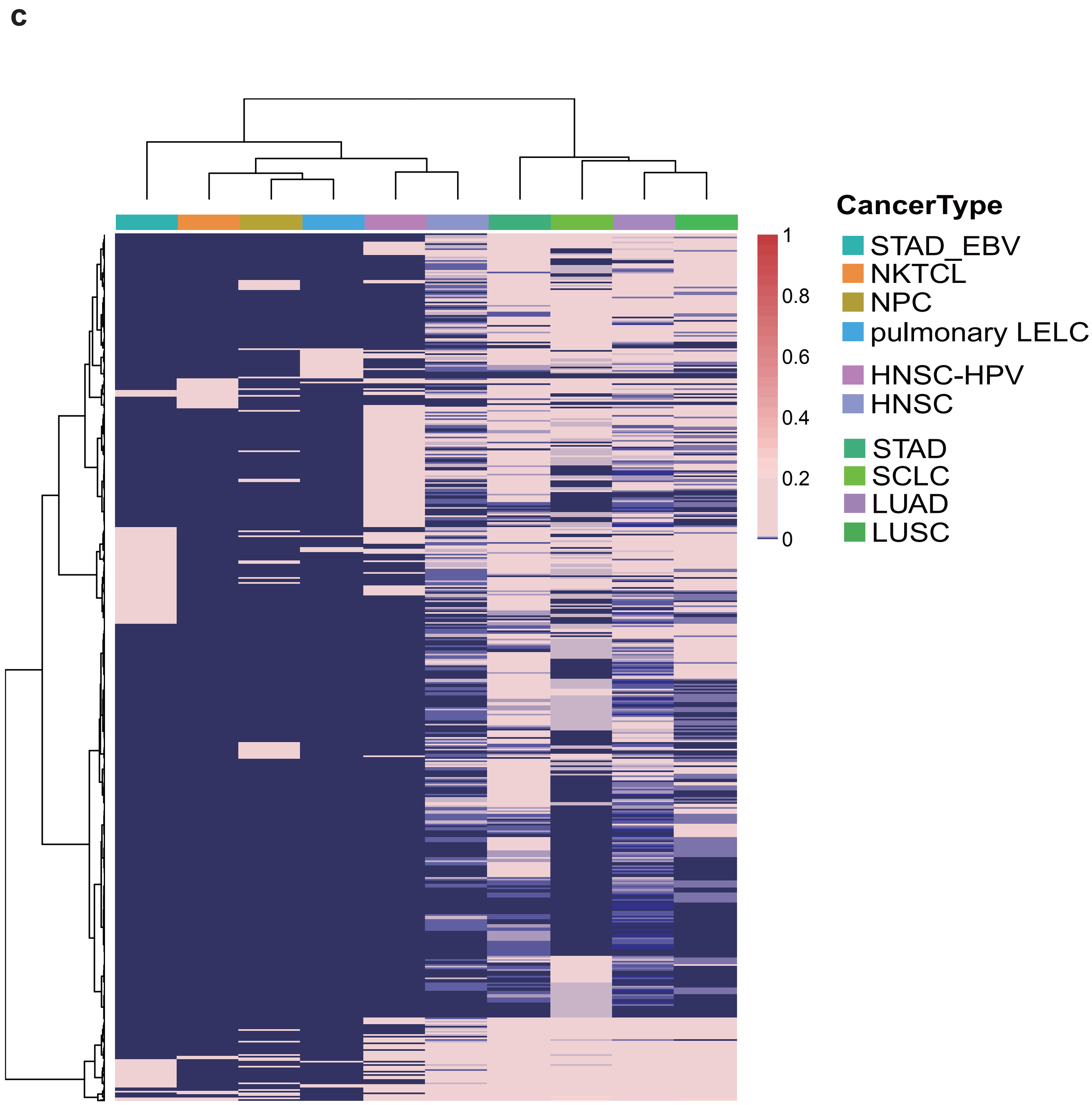

Supplementary Figure 12. Mutation spectrum comparison between pulmonary LELC and other cancer types. a, Pearson correlation of mutation spectrum (six mutation types of non-silent SNVs) in all cancer types. **b,** Unsupervised hierarchical clustering of 96 subtypes based on six mutation types and nucleotides flanking the mutated base for all cancer types. **c,** Unsupervised hierarchical clustering of mutated gene frequency for all cancer types. NPC, nasopharyngeal carcinoma; LELC, lymphoepithelioma-like carcinoma; NKTCL, natural killer/T cell lymphoma; HNSC, head and neck squamous cell carcinoma; HPV, human papillomavirus; LUAD, lung adenocarcinoma; LUSC, lung squamous cell carcinoma; SCLC, small-cell lung carcinoma; STAD, stomach adenocarcinoma; SNV, single nucleotide variants.

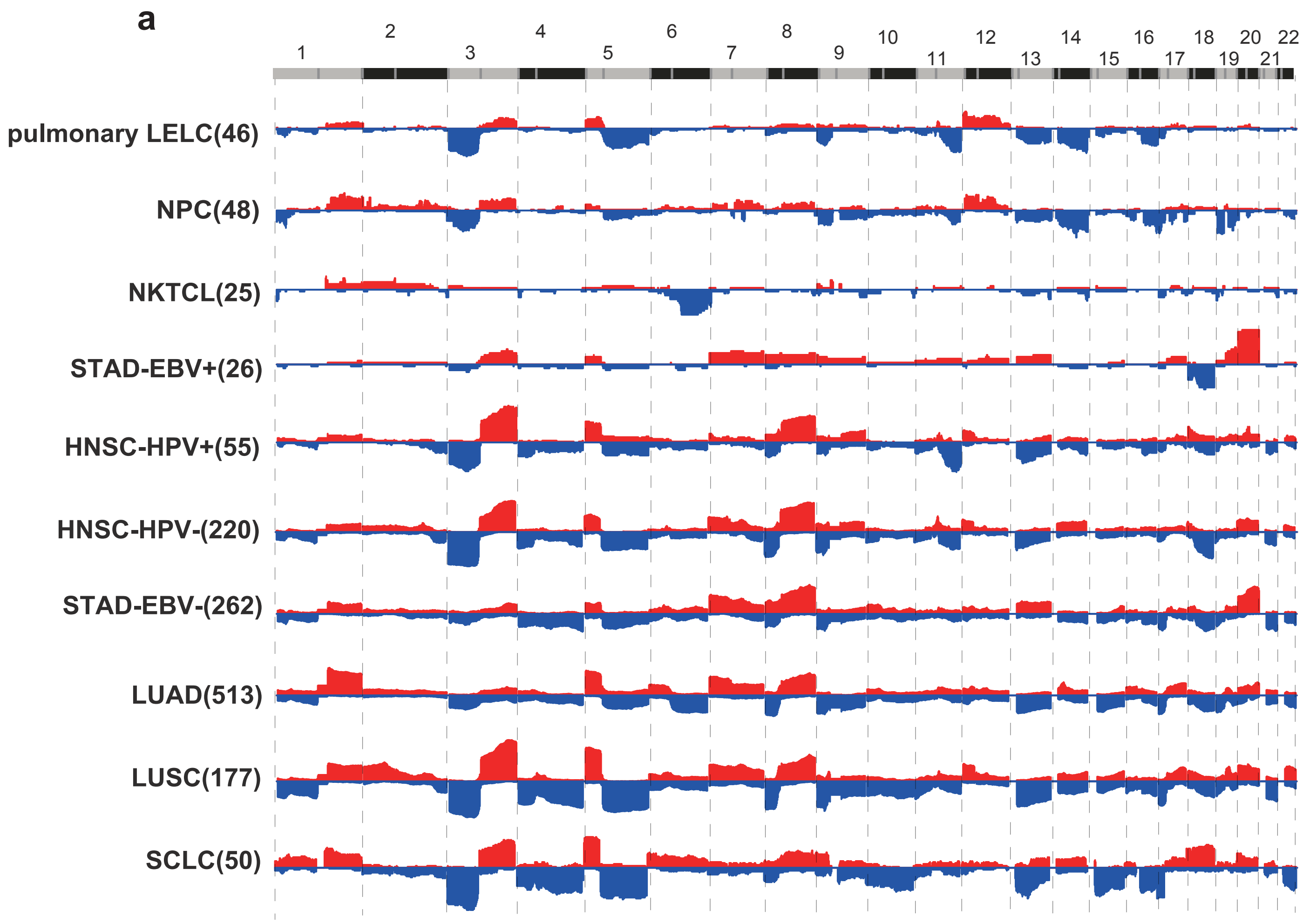
a
1
2
3
4
5
6
7
8
9
10
11
12
13
14
15
16
17
18
19
20
21
22
pulmonary LELC(46)
NPC(48)
NKTCL(25)
STAD-EBV+(26)
HNSC-HPV+(55)
HNSC-HPV-(220)
STAD-EBV-(262)
LUAD(513)
LUSC(177)
SCLC(50)

b

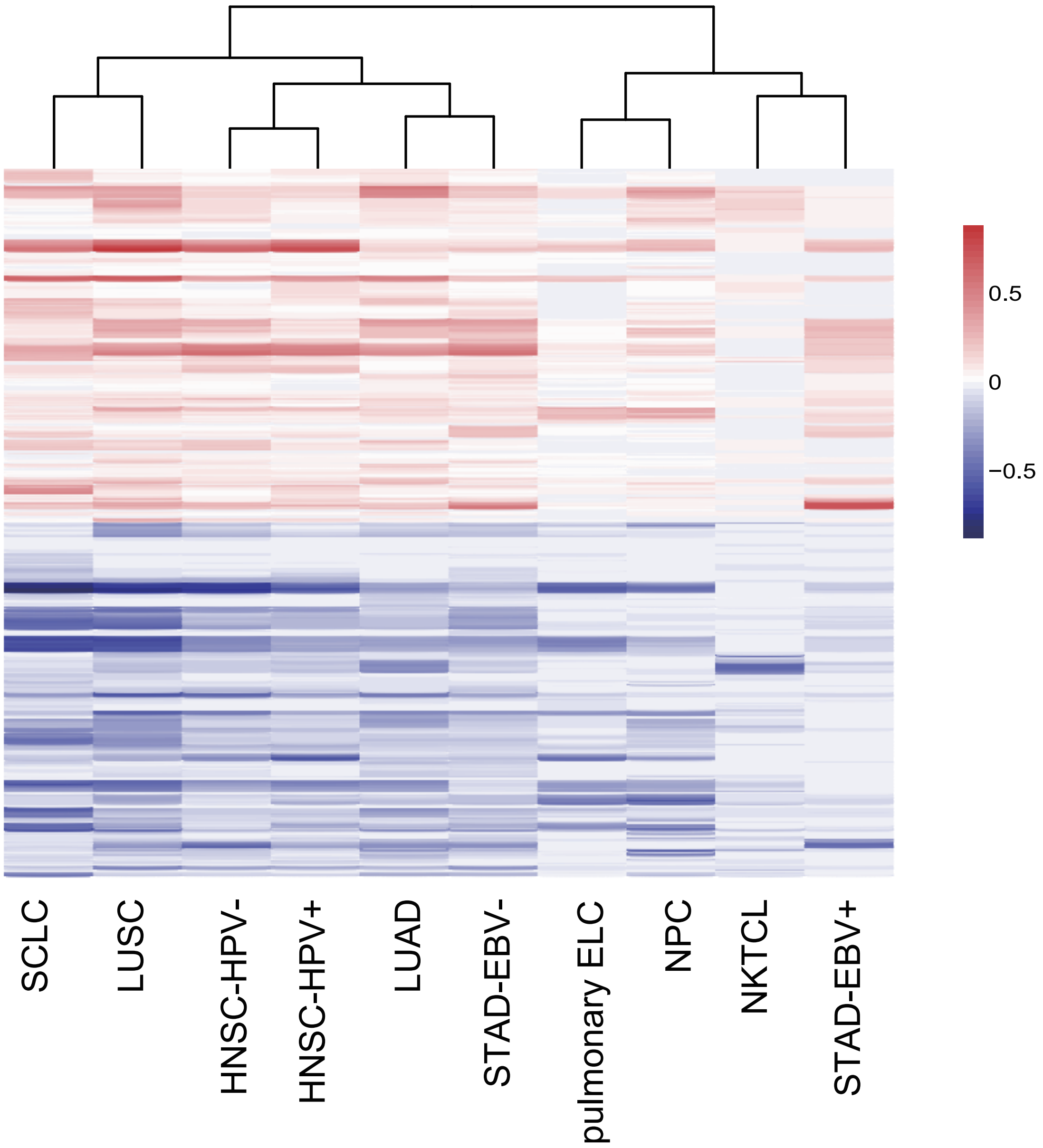

Supplementary Figure 13. Comparison of copy number alterations between pulmonary LELC and other cancer types. a, Copy number alteration frequency for all cancer types with gains in red and losses in blue. **b,** Unsupervised hierarchical clustering of copy number alterations frequency for all cancer types. LELC, LELC, lymphoepithelioma-like carcinoma; NPC, nasopharyngeal carcinoma; NKTCL, NKT cell lymphoma; STAD, stomach adenocarcinoma; HNSC, head and neck squamous carcinoma; HPV, human papillomavirus; LUAD, lung adenocarcinoma; LUSC, lung squamous carcinoma; SCLC, small-cell lung carcinoma.

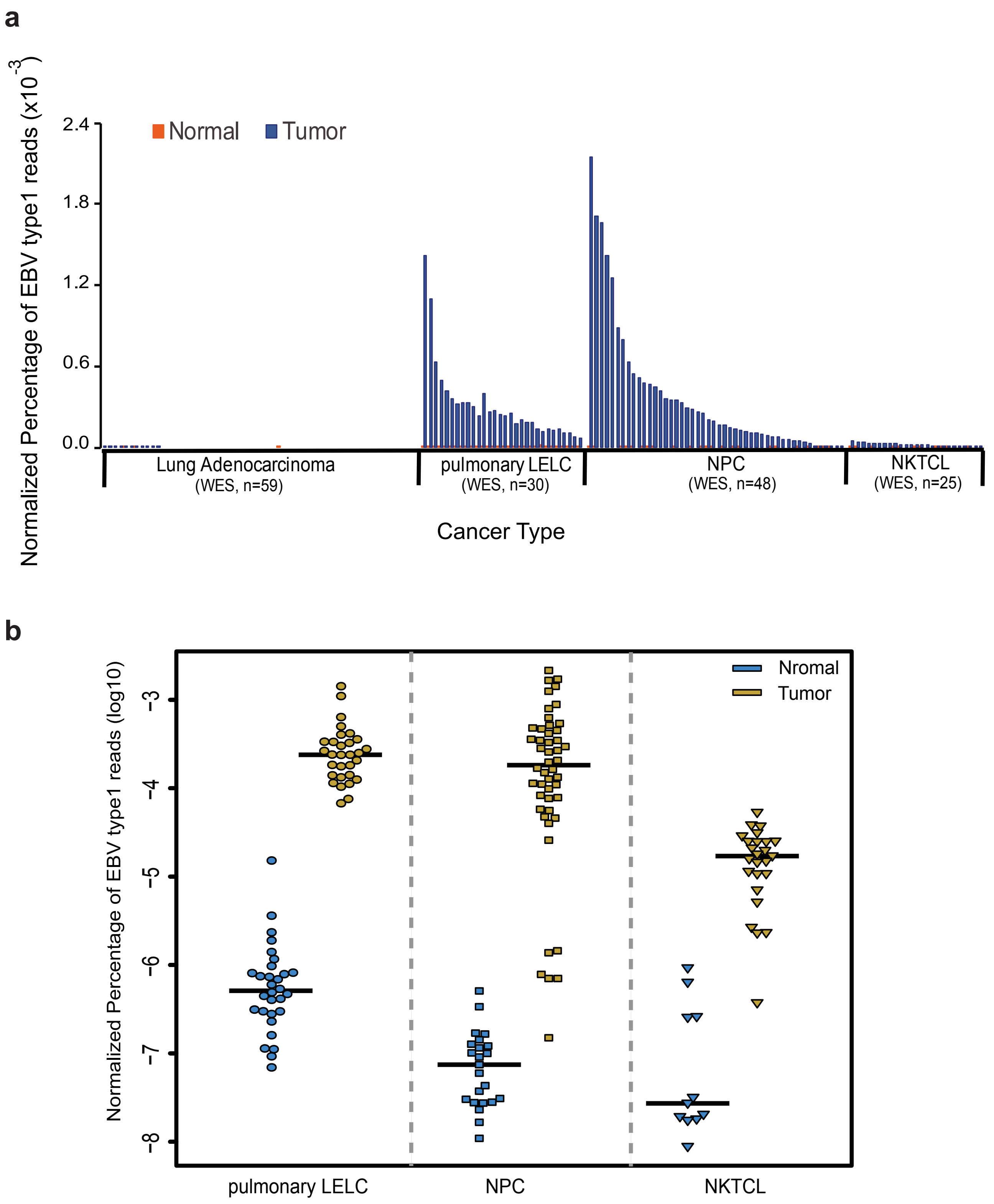

Supplementary Figure 14 . Comparison of EBV sequences between pulmonary LELC, NPC and NKTCL. a, Distribution of the percentage for EBV sequences which is calculated by (reads mapped to the EBV type 1 genome)/(total sequencing reads) in tumor and matched normal samples from lung adenocarcinoma, pulmonary LELC, NPC and NKTCL. **b,** Plot for the percentage of EBV sequences normalized by log10. Bold black lines in the scatter plot of **b** denote the median value. EBV, Epstein-Barr virus; LELC, lymphoepithelioma-like carcinoma; NPC, nasopharyngeal carcinoma; NKTCL, NKT cell lymphoma; WES, whole exon sequencing.

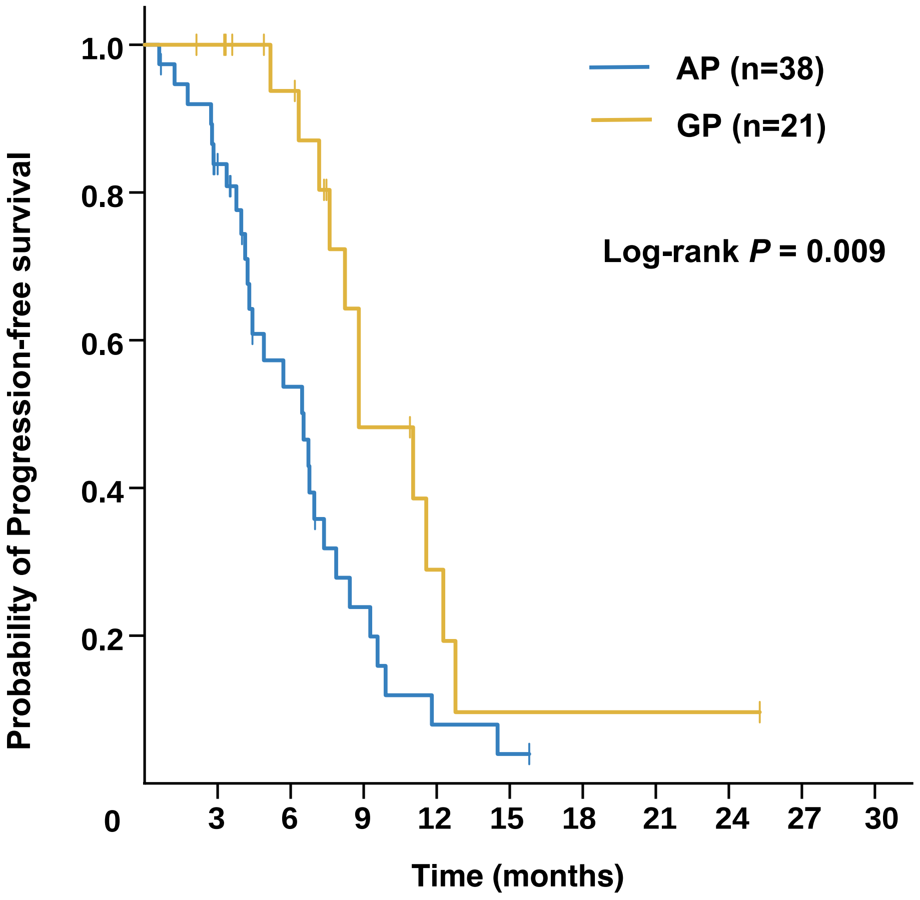

Supplementary Figure 15 . Progression-free survival of metastatic pulmonary LELC patients recieving first-line chemotherapy of pemetrexed plus platinum (AP) or gemcitabine plus platinum (GP).

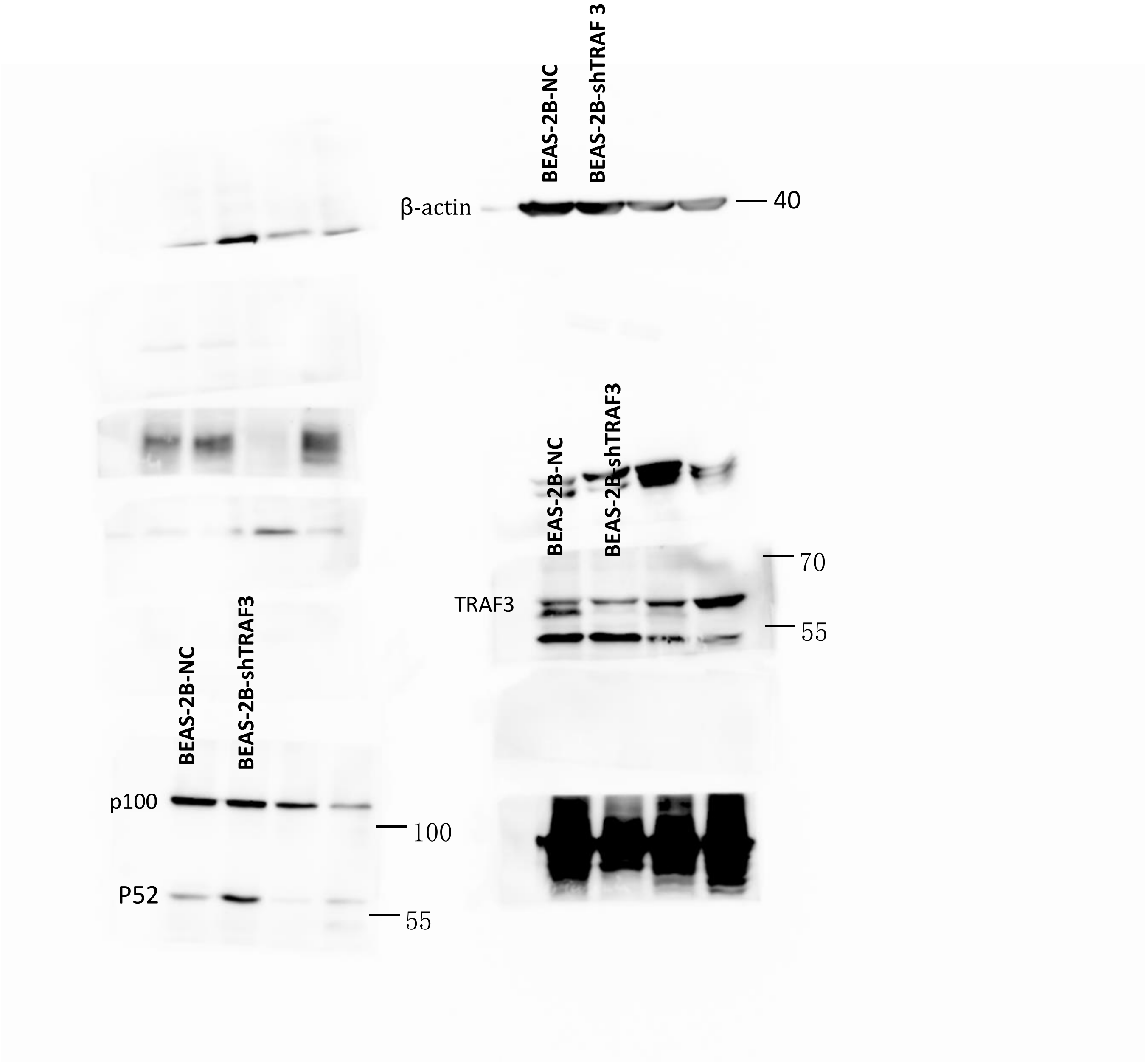

Supplementary Figure 16 . Uncropped and unprocessed scans for Fig. 4c.

Supplementary Figures References

1. Lin, D. C. *et a*l. Genomic and molecular characterization of esophageal squamous cell carcinoma. *Nat Genet* 46, 467-473, doi:10.1038/ng.2935 (2014).

2. Hua X, Hyland PL, Huang J, *et a*l. MEGSA: A Powerful and Flexible Framework for Analyzing Mutual Exclusivity of Tumor Mutations. *Am J Hum Genet* 2016;98(3): 442-455.
