# Supplementary material for: The genomic landscape of Epstein-Barr virus-associated pulmonary lymphoepithelioma-like carcinoma
Source: Nat Commun. 2019 Jul 16;10:3108. doi: 10.1038/s41467-019-10902-w (PMC6635366; doi:10.1038/s41467-019-10902-w)
Supplement: Supplementary file 3 — Description of Additional Supplementary Information [file 41467_2019_10902_MOESM3_ESM.docx]

**Description of Additional Supplementary Files**

File Name: Supplementary Data 1
Description: Clinicopathological characteristics of 91 pulmonary lymphoepithelioma-like carcinomas (LELC) patients

File Name: Supplementary Data 2

Description: Clinical feature summary of 91 pulmonary lymphoepithelioma-like carcinomas (LELC)

File Name: Supplementary Data 3
Description: Summary statistics of sequencing data from 91 pulmonary lymphoepithelioma-like carcinomas (LELC) patients

File Name: Supplementary Data 4
Description: Somatic mutation of 30 pair pulmonary LELC samples in exon of whole-exon sequencing

File Name: Supplementary Data 5
Description: Somatic mutation of 61 pulmonary LELC tumor samples of target deep sequencing

File Name: Supplementary Data 6
Description: Verification of somatic mutations by target deep sequencing on 53 genes across 29 pulmonary LELC tumors

File Name: Supplementary Data 7
Description: Somatic mutation sites number of each gene for 114 selected genes form 91 pulmonary LELC samples

File Name: Supplementary Data 8
Description: GSEA pathway enrichment analysis for genes with copy number gain in chromosome 12

File Name: Supplementary Data 9
Description: Focal copy number alterations identified by GISTIC2.0 analysis of 46 pulmonary LELC tumor samples

File Name: Supplementary Data 10
Copy number alteration number of each gene for 46 pulmonary LELC tumors (genes were ordered according to descending frequency of deletion)

File Name: Supplementary Data 11
Description: Association between the three core signaling pathway alterations and LMP1 overexpression

File Name: Supplementary Data 12
Description: Clinicopathological characteristics of 59 metastatic pulmonary lymphoepithelioma-like carcinomas (LELC) patients who received pemetrexed plus platinum (AP) or gemcitabine plus platinum (GP) as first-line treatment

File Name: Supplementary Data 13

Description: Multivariate analysis of the progression-free survival and objective response rate for patients with advanced pulmonary lymphoepithelioma-like carcinomas (LELC)
